# Multi-tissue RNA-Seq Analysis and Long-read-based Genome Assembly Reveal Complex Sex-specific Gene Regulation and Molecular Evolution in the Manila Clam

**DOI:** 10.1093/gbe/evac171

**Published:** 2022-12-12

**Authors:** Ran Xu, Jacopo Martelossi, Morgan Smits, Mariangela Iannello, Luca Peruzza, Massimiliano Babbucci, Massimo Milan, Joseph P Dunham, Sophie Breton, Liliana Milani, Sergey V Nuzhdin, Luca Bargelloni, Marco Passamonti, Fabrizio Ghiselli

**Affiliations:** Department of Biological, Geological, and Environmental Sciences, University of Bologna, Bologna, Italy; Department of Biological, Geological, and Environmental Sciences, University of Bologna, Bologna, Italy; Department of Comparative Biomedicine and Food Science, University of Padova, Padova, Italy; Department of Biological, Geological, and Environmental Sciences, University of Bologna, Bologna, Italy; Department of Comparative Biomedicine and Food Science, University of Padova, Padova, Italy; Department of Comparative Biomedicine and Food Science, University of Padova, Padova, Italy; Department of Comparative Biomedicine and Food Science, University of Padova, Padova, Italy; Program in Molecular and Computational Biology, University of Southern California, Los Angeles, CA, USA; SeqOnce Biosciences Inc., Pasadena, CA, USA; Department of Biological Sciences, University of Montreal, Montreal, Canada; Department of Biological, Geological, and Environmental Sciences, University of Bologna, Bologna, Italy; Program in Molecular and Computational Biology, University of Southern California, Los Angeles, CA, USA; Department of Comparative Biomedicine and Food Science, University of Padova, Padova, Italy; Department of Biological, Geological, and Environmental Sciences, University of Bologna, Bologna, Italy; Department of Biological, Geological, and Environmental Sciences, University of Bologna, Bologna, Italy

**Keywords:** long-read genome assembly, differential transcription, co-expression network, alternative splicing, tissue specificity, sexual contrasting genetic markers

## Abstract

The molecular factors and gene regulation involved in sex determination and gonad differentiation in bivalve molluscs are unknown. It has been suggested that doubly uniparental inheritance (DUI) of mitochondria may be involved in these processes in species such as the ubiquitous and commercially relevant Manila clam, *Ruditapes philippinarum*. We present the first long-read-based de novo genome assembly of a Manila clam, and a RNA-Seq multi-tissue analysis of 15 females and 15 males. The highly contiguous genome assembly was used as reference to investigate gene expression, alternative splicing, sequence evolution, tissue-specific co-expression networks, and sexual contrasting SNPs. Differential expression (DE) and differential splicing (DS) analyses revealed sex-specific transcriptional regulation in gonads, but not in somatic tissues. Co-expression networks revealed complex gene regulation in gonads, and genes in gonad-associated modules showed high tissue specificity. However, male gonad-associated modules showed contrasting patterns of sequence evolution and tissue specificity. One gene set was related to the structural organization of male gametes and presented slow sequence evolution but high pleiotropy, whereas another gene set was enriched in reproduction-related processes and characterized by fast sequence evolution and tissue specificity. Sexual contrasting SNPs were found in genes overrepresented in mitochondrial-related functions, providing new candidates for investigating the relationship between mitochondria and sex in DUI species. Together, these results increase our understanding of the role of DE, DS, and sequence evolution of sex-specific genes in an understudied taxon. We also provide resourceful genomic data for studies regarding sex diagnosis and breeding in bivalves.

**Significance**

The Manila clam displays many interesting biological features such as annual renewal of gonads, sex-changing capability during life cycle, and two segregated mitochondrial lineages with sex-specific transmission (doubly uniparental inheritance). Sex-specific gene expression and sequence evolution have been explored mainly in organisms with sexual dimorphism, and they remain largely understudied in bivalves, which lack heteromorphic sex chromosomes and secondary sexual characters. Through tissue-specific transcriptome analysis, we highlighted gene regulation patterns in gonads: male-gonad-associated genes showed two distinct patterns of sequence evolution and tissue specificity, probably due to the functional differences in the corresponding gene sets. This work improves our understanding of tissue and sex-specific gene regulation and evolution in bivalves.

## Introduction

Bivalves show an astonishing wealth of diverse life histories, adaptation, and phenotypic plasticity. Numerous species have become important biological models for monitoring pollution, studying adaptation to climate change, and developing biomedical tools (Krishnakumar et al. 2018; Harris et al. 2020). Moreover, many bivalves have a global economic importance, providing an essential source of protein through aquaculture and fishing (Wijsman et al. 2019).

Despite their important ecological and economic roles, and their biodiversity within Mollusca phylum, bivalves (and Molluscs in general) have been poorly investigated at the molecular level, compared to other animal groups. This is even more surprising if we consider that bivalves show peculiar biological features which make them ideal model systems in fields like evolutionary, molecular, and developmental biology (Ghiselli et al. 2021a). Bivalvia present a variety of sexual reproduction modes, ranging from strict gonochorism to sequential or simultaneous hermaphroditism (Breton et al. 2018). So far, no heteromorphic sex chromosomes have been found in bivalves and the molecular factors involved in sex determination and gonad differentiation are unknown: it has been proposed that the variety in reproduction modes is primarily due to modifications of the same genetic pathways (Breton et al. 2018). Given this context, investigating tissue-specific gene regulation may help identify gene networks involved in sex determination and gonad differentiation. In other animal species investigated so far, regulation of gene transcription, in terms of differential expression (DE) and differential splicing (DS), is known to be involved in resolving sexual conflicts (Ingleby et al. 2015; Ghiselli et al. 2018; Rogers et al. 2021). Indeed, most of the sex-specific characters are the result of genes that are differentially expressed between sexes (sex-biased genes), and rapid sequence evolution of sex-biased genes has been observed in animals (Ellegren and Parsch, 2007; Mank et al. 2007; Harrison et al. 2015; Lipinska et al. 2015; Dean and Mank, 2016; Ghiselli et al. 2018). Additionally, several studies revealed that a large proportion of genes undergo sex-specific splicing, indicating a role of DS in sex-specific development and physiology (Telonis-Scott et al. 2009; Griffin et al. 2013; Rogers et al. 2021). In species with sexual dimorphism, sexual selection was suggested as a driver of sex-biased patterns of gene expression and splicing, whereas gene expression breadth, protein–protein interaction, codon usage, and pleiotropy may also contribute to sex bias (Mank et al. 2008; Harrison et al. 2015; Grath and Parsch, 2016; Whittle and Extavour, 2019). If and how these factors shape the evolution of species lacking sexual dimorphism, as in the case of most bivalves, has yet to be explored.

Another interesting feature, found in more than 100 bivalve species, is the presence of the doubly uniparental inheritance (DUI) of mitochondria. In DUI species, two distinct lineages of mitochondrial DNA (mtDNA) are inherited by the offspring: one lineage (F-type) is transmitted through eggs and it is present in both sexes, the other (M-type) is transmitted through sperm and it is mainly found in males (less often in females and in lower abundance) where it is most abundant in gonads (Ghiselli et al. 2019; Ghiselli et al. 2021b). Numerous works have sought to elucidate the molecular mechanisms beyond DUI and speculated on the evolutionary process behind the maintenance of divergent mtDNA lineages within species, especially considering that heteroplasmy is generally considered an unfavorable condition, generally converging on the hypothesis that DUI might be linked to sexual differentiation (Breton et al. 2018; Capt et al. 2018). Having two different mitochondrial genomes with sex-specific and tissue-specific distribution opens up questions about the existence of tissue and sex-specific coordination of gene regulation, namely regarding nuclear genes involved in mitochondrial biology (Ghiselli et al. 2021b; Maeda et al. 2021; Xu et al. 2022).

In this work, we performed a de novo long-read genome assembly and a multi-tissue RNA-seq analysis of *Ruditapes philippinarum*, a gonochoric bivalve species with DUI, to investigate sex-specific and tissue-specific gene regulation and molecular evolution. More in detail, we compared differential gene transcription and DS across tissues for the first time in bivalves, focusing on differences between somatic tissues and gonads. We also investigated the relationship between tissue-specific co-expressed modules and protein sequence evolution. Our aim was to identify genes and gene networks that could have a major role in tissue differentiation, and characterize their patterns of evolution. We found that gonads, compared to somatic tissues, show a more complex gene regulation, as multiple co-expression submodules are present within the same tissue. Some of these submodules are also sex-specific, showing peculiar and divergent patterns of sequence evolution. We finally identified hub genes for each tissue-specific module, which are likely to be crucial for tissue specification, and we highlighted those that could have a central role in sex determination/differentiation and those that could have a possible role in DUI.

## Results

### Genome Sequencing, De Novo Assembly, and Whole-Genome Alignment

PacBio sequencing consisted of 54 SMRT cells that yielded ∼4 M reads (36.5 Gb) of raw sequences with a median length of ∼45 Kb. The Illumina sequencing resulted in ∼145 M reads (∼75 Gb) for the short insert library, and ∼48 M reads (∼25 Gb) for the long insert library. After trimming both Illumina libraries, a total of ∼180 M PE reads were kept (supplementary table S1, Supplementary Material online).

We estimated a genome size of ∼1.37 Gb (supplementary table S2, Supplementary Material online) giving an expected genome coverage of ∼25 × and ∼72 × for, respectively, the PacBio and Illumina libraries. The estimated genome size resulted concordant with previous kmer-based estimations which range from 1.32 Gb (Yan et al. 2019) to 1.37 Gb (Mun et al. 2017), but quite smaller from than the 1.97 Gb estimation obtained by the Feulgen method (González-Tizón et al. 2000). The heterozygosity and the repetitive content were estimated to range, respectively, from 4% to 3.7% and from 61.2% to 48.2%, depending on the kmer size (supplementary table S3 and supplementary fig. S1, Supplementary Material online). After three rounds of purging and polishing, the final version of the assembly consisted in 15,908 contigs with a N50 of 183 Kb, a total genome size of 1.41 Gb and a mean GC content of 0.32. We identified 884 out of 954 Metazoa BUSCO orthologs (92.7%), of which 802 were present as single copy (84.1%), and 82 as duplicates (8.6%). Missing genes represent 4.7% of the core gene set, whereas only 2.6% were identified as fragmented (table 1). KAT analyses show a kmer completeness of 52,48% (table 1; supplementary fig. S2, Supplementary Material online), and 95% and 98% of the short and long reads were successfully remapped on the assembly, respectively, with a median coverage depth of 53.42 and 22.69 (table 1). Blobtools identified 20 contigs as possible bacterial contaminations. Another six contigs were annotated as belonging to Priapulida, whereas only one to Zoopagomycota. These contigs cover 937,293 bp of the total assembly size (0.0007%) and were removed from the final version of the assembly.

**Table 1:** Summary Statistics of the Long-Read-based Manila Clam Assembly

Assembly genome size	1,409,123,410 bp
Number of contigs	15,908
Average contig length	88,579.55 bp
Largest contig	1,574,940 bp
L50	2,143 bp
N50	182,737 bp
N90	37,082 bp
BUSCO	C:92.7% [S:84.1%, D:8.6%], F:2.6%, M:4.7%, *n*:954
Mapped short reads	343,975,629 (95%)
Mapped long reads	12,691,865 (98%)
Median short reads depth	53.42
Median long reads depth	22.69
Kmer completeness	52,48%
GC content	0.32

For a direct comparison between our newly produced assembly and the short-reads-only chromosome-level assembly from Yan et al. (2019), from now on “CRph genome” we performed a pairwise whole-genome alignment (WGA). Out of the 15,908 contigs that composed our assembly, 99.2% had at least one alignment block to the CRph genome with the majority of alignments involving an assembled chromosome. In total, all alignment blocks represented 80% of our assembly and 77.4% of the CRph genome (supplementary table S4, Supplementary Material online). Detailed results and discussion for genome assembly and comparison can be found in Supplementary Materials, Methods and Results, Supplementary Material online.

### Genome Annotation

Using de novo approaches, we built up a starting consensus library composed of 5,600 sequences (3,197 and 2,403 by RepeatModeler and MITE_Tracker, respectively). We added another 1,031 TEs already characterized in molluscs and retrieved from RepBase. After removal of genes/gene fragments, tandem, and low copy number repeats (<5 good hits on the genome), we used a total of 2,332 nonredundant consensus sequences to annotate the *R. philippinarum* repeatome. Overall, 39.7% of the genome was masked by interspersed repeats with a prevalence of cut and paste (DNA + MITEs) and Rolling Circle TEs (14.7% Unknown elements; 9.23% MITEs; 6.1% Rolling circle; 3.5% DNA; 2.95% LINE; 1.84% LTR; 1.25% SINE) (supplementary table S5, Supplementary Material online).

The annotation pipeline generated 34,505 gene models with an average length of 8,053 bp (6.4 mean exons per gene; mean exon length: 212 bp). Of these, 22,103 (64%) had a positive match by blastx against the Swiss-prot database (supplementary table S5, Supplementary Material online). The Annotation Edit Distance (AED), a metric useful to measure the agreement between predicted gene models and external evidence, where a value of 0 indicates full agreement and 1 no external support (Holt, 2011), identified 29,322 (85%) gene models with an AED ≤0.5 and a mean equal to 0.18. BUSCO scores on the predicted proteomes using the Metazoa odb10 reference database resulted in C:83.4%[S:74.5%, D:8.9%], F:8.8%, M:7.8%. The percentage of RNA-seq reads mapped to the genome is reported in supplementary table S6, Supplementary Material online.

### Differential Expression and Co-Expression Network

**Fig. 1.**
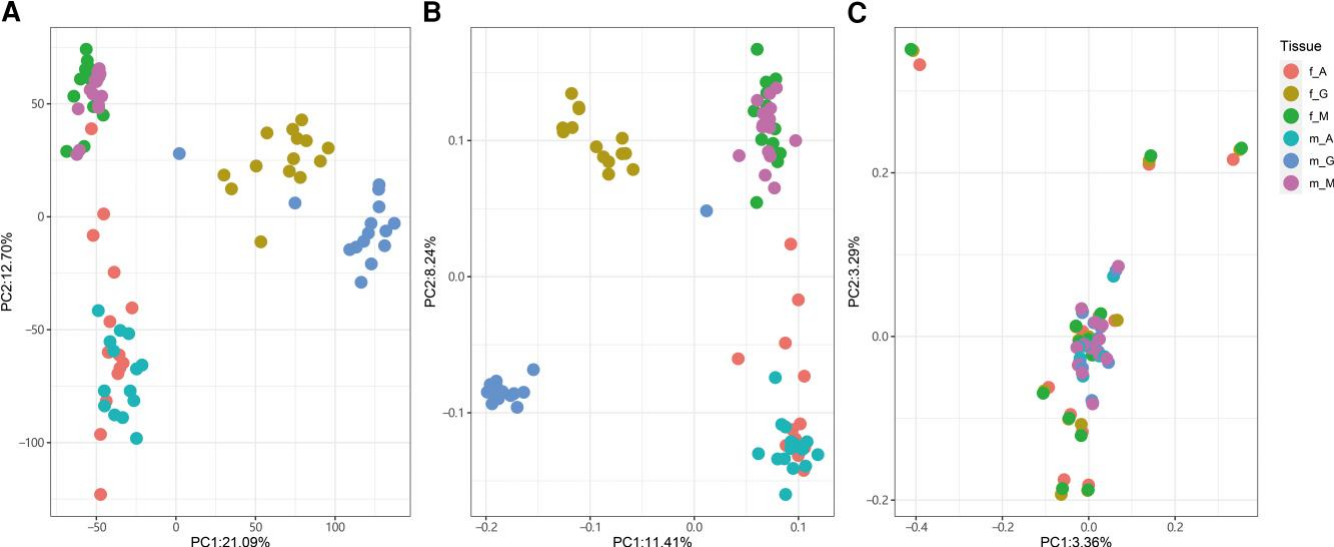
PCA plot for gene expression (*A*), alternative splicing (*B*), and genotype (*C*). Each dot represents a sample and each color represents a tissue type; f_A: female adductor; f_G: female gonad; f_M; female mantle; m_A: male adductor; m_G: male gonad; m_M: male mantle.

**Fig. 2.**
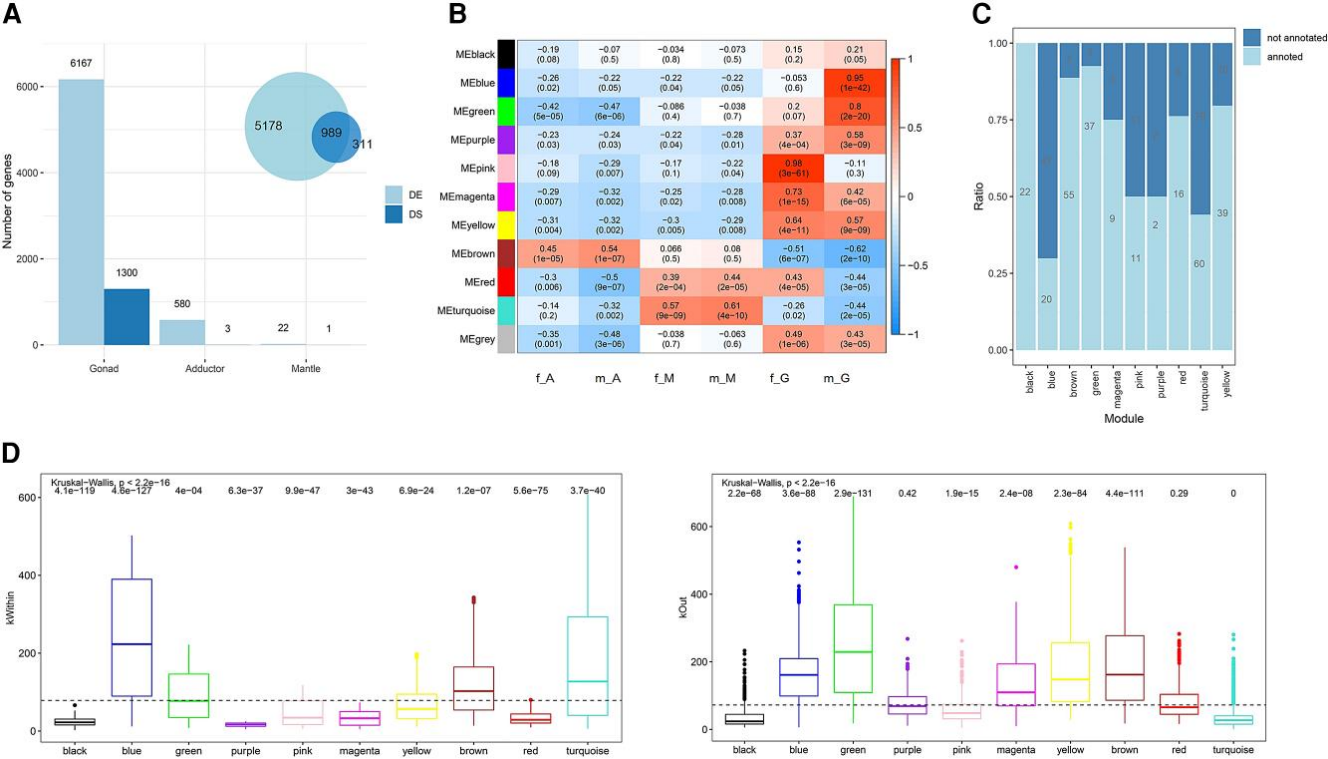
Differentially expressed, spliced and co-expressed genes across tissue types. (*A*) The number of differentially expressed (DE) and spliced (DS) genes between females and males in each tissue. The Venn plot on the top-left represents the overlap between DE and DS genes in the gonad. (*B*) Module-tissue association based on the gene expression. Each row corresponds to a co-expression module and the module name is shown on the left. Each column represents a tissue type. The correlations and *P* values between module and tissue are shown in each cell. (*C*) The proportion of annotated and not annotated hub genes in each module. Numbers in the bars indicate the number of hub genes for each module. (*D*) The distribution of within (kWithin) module connectivity and outside (kOut) module connectivity for genes in the co-expression modules. Wilcoxon rank-sum test with FDR corrections was used to compare the distribution of kWithin and kOut in each module to the overall distribution and the significance were shown on the top of the boxplot. ***, *P* < 0.0001; **, *P* < 0.001; *, *P* < 0.05; ns, non-significant. The dash line indicates the median of the overall distribution.

To investigate the global expression patterns in all tissues of both sexes, a PCA analysis was performed in DESeq2. As shown in figure 1*A*, different tissues presented distinct expression profiles, and while expression patterns between female and male somatic tissues were quite similar, large differences were found in gonads. Consistently, the number of differentially expressed genes (DEGs) between female and male adductor muscles and mantles were low (578 and 22, respectively), whereas the number of DEGs between gonads were 6,167, including 3,024 female-biased DEGs and 3,143 male-biased DEGs (fig. 2*A*). The comparisons of DEGs between pairwise tissues were performed for males and females separately. Generally, the number of DEGs between somatic tissues (adductor muscle vs. mantle) was less than the number of DEGs between somatic tissue and gonad (e.g., gonad vs. mantle) (supplementary table S7, Supplementary Material online). A large proportion of DEGs in females in pairwise tissue comparisons overlapped with the corresponding DEGs in males (supplementary table S8, Supplementary Material online). In all pairwise tissue comparisons, 1,787 and 2,277 genes were differentially expressed across all three tissues in females and males, respectively (supplementary fig. S3*A*, Supplementary Material online), and 1,009 of these DEGs were shared between females and males. Additionally, to investigate the genes showing significant sex-by-tissue interactions, we performed a DE analysis using Likelihood ratio test. The number of genes across tissues, between sexes, and between sexes across tissues was 17,802, 6,321, and 4,430, respectively.

**Fig. 3.**
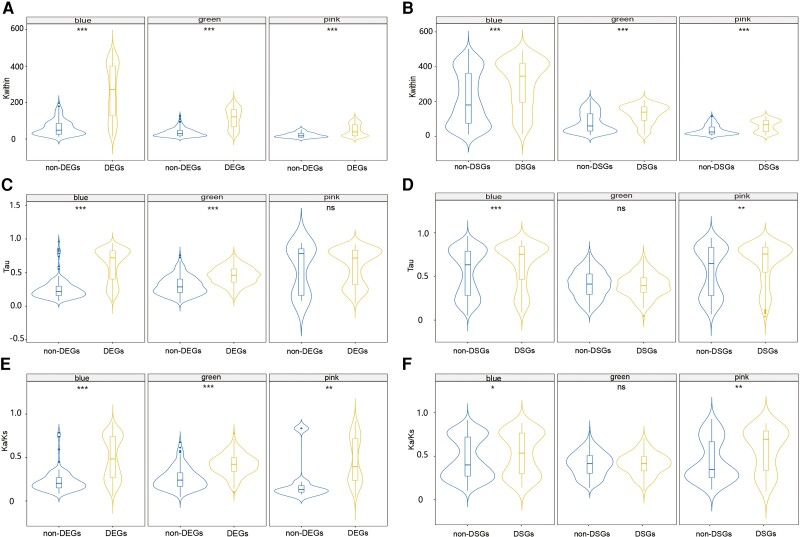
Comparisons between differentially expressed genes (DEGs) and non-DEGs, and between differentially spliced genes (DSGs) and non-DSGs in female and male gonad-associated modules. (*A*), (*C*), and (*E*) represent comparisons of the connectivity, tissue specificity, and sequence evolutionary rate between DEG and non-DEGs. (*B*), (*D*), (*F*) represent the comparisons of the connectivity, tissue specificity, and sequence evolutionary rate between DSGs and non-DSGs. The module name is shown on the top of each panel.

A tissue-specific gene co-expression network was constructed to investigate gene regulatory relationships in tissue-associated modules. A total number of 8,640 genes were assigned to 10 modules (fig. 2*B*). The blue module (1,334 genes) and the green module (790 genes) showed high association with male gonads, whereas the pink module (417 genes) was associated with female gonads. Moreover, yellow (977 genes), magenta (232 genes), and purple (80 genes) modules were associated with both female and male gonads, and turquoise (2,718 genes) and brown (1,749 genes) were associated with somatic tissues. Moreover, we retrieved “hub” genes which rank in the top 5% of kWithin in each module and represent high connection with the other genes. Hub genes and functional annotations in each module are listed in supplementary table S9, Supplementary Material online. We found that the percentage of hub genes with annotation varied across modules (fig. 2*C*). These genes included male-gonad-specific *SRY-box transcription factor 30* (*sox30*) in the male-gonad-specific blue module, and *mating-type-like protein ALPHA2* (*mtlalpha2*) in the female-gonad-specific pink module. For genes in the co-expression network, we measured the connectivity among genes in the same module (intramodular connectivity: kWithin), the connectivity between genes from different modules (intermodular connectivity: kOut), and the global connectivity (kTotal = kWithin + kOut). In this tissue-specific co-expression network, kWithin represents within module connectivity specific to one or multiple associated tissue types (specific connectivity), whereas kOut represents the connectivity of one gene to the genes outside the module in the other tissue types (broad connectivity). The distribution of intramodular connectivity (kWithin) and intermodular connectivity (kOut) for genes in each module is shown in figure 2*D*, and the statistical tests for pairwise comparisons of connectivity between modules are shown in supplementary table S10, Supplementary Material online. Generally, the gonad-associated blue module and mantle-associated turquoise module presented significantly higher kWithin compared with the overall distribution, whereas another gonad-associated green module presented significantly higher kOut (fig. 2*D*). A predominant number of 1,253 (93.9%), 579 (73.3%), and 397 (95.2%) genes in the blue, green, and pink modules, respectively, were also DEGs between female and male gonads. Besides, the kWithin for DEGs in gonad-associated blue, green, and pink modules were significantly higher than non-DEGs between female and male gonads (fig. 3*A*).

A GO enrichment analysis was applied to explore the predicted functions of different subsets of genes, and the results are shown in supplementary table S11, Supplementary Material online. Considering the low number of DEGs between female and male mantles, we did not perform the enrichment analysis on this subset of genes. The significantly enriched GO terms in adductor muscles between males and females were related to microtubule-based process and motor activity (supplementary table S11, Supplementary Material online). Reproduction and cell cycle-related processes were significantly enriched for the DEGs between female and male gonads, and for the DEGs between sexes across tissues (supplementary table S11, Supplementary Material online). Reproduction-related processes were also enriched in the male gonad-associated blue module (supplementary table S11, Supplementary Material online). Notably, Kelch-related domains were significantly overrepresented in the blue module (supplementary table S12, Supplementary Material online). The genes co-expressed in the other male gonad-associated (green) module appeared to over-represent some general functions, with processes like “organelle assembly”, “cell project”, and “catalytic activity” being enriched. In the female gonad-associated pink module, processes related to transferase and protein metabolic activities, and homeobox-related domains were significantly enriched (supplementary table S11 and S12, Supplementary Material online). Different functional processes were enriched in the three gonad-associated modules (magenta, purple, and yellow) such as “cell adhesion” (purple and magenta modules), homeostatic related processes (purple module), and processes related to tissue development (magenta module) (supplementary table S11, Supplementary Material online). Intriguingly, for genes in the yellow module, processes related to DNA repair, DNA replication, and gene expression were significantly enriched. In mantle-associated turquoise modules, genes were overrepresented in the immune-related process and metal ion binding.

### Differential Splicing Analysis

Consistent with expression profiles, splicing patterns also differed across tissues, and differences between females and males were observed in gonads but not in somatic tissues (fig. 1*B*). Global alternative splicing events for each tissue are shown in supplementary fig. S4, Supplementary Material online. Generally, skipping exon (SE), alternative 5′ splicing (A5), and alternative first exon (AF) accounted for a large proportion in all tissues, while retained intron (RI) and mutually exclusive exons (MXE) were the least represented events in all tissues. Moreover, alternative splicing in gonads and mantles seemed to be more frequent than in adductor muscles. Despite the pervasiveness of alternative splicing in all tissues, the number of genes showing DS between females and males in each tissue, and between pairwise tissues were far less compared with DEGs. The number of differentially spliced genes (DSGs) between female and male adductor muscles, mantles, and gonads were 3, 1, and 1,300, respectively (fig. 2*A*). Notably, among all the 1,300 DSGs between female and male gonads, 989 (76%) were also differentially expressed between female and male gonads. We also retrieved these DSGs in three sex-associated co-expression modules (blue, green, and pink) and we found that the DSGs in these modules showed significantly higher kWithin than non-DSGs (fig. 3*B*). The number of DSGs between gonads and somatic tissues was higher than that found between two somatic tissues (supplementary fig. S3 and supplementary table S6, Supplementary Material online). Moreover, in all these comparisons between different tissues, DEGs and DSGs were largely overlapping for both females and males, and around 80–90% DSGs between gonads and somatic tissues were also DEGs (supplementary table S8, Supplementary Material online). Some of these DSGs overlapped with DEGs or sex-associated modules (listed in supplementary table S9, Supplementary Material online), and the large amount of overlapping genes between DSGs and DEGs in gonads also resulted to have many processes in common, such as “microtubule-based process” and “cellular process”. Additionally, functional characterization of DSGs that did not overlap with DEGs, highlighted their involvement in chromatin remodeling and mRNA catabolic processes.

### Tissue Specificity in the Co-Expression Network

The tissue specificity index Tau ranged from 0.2 to 0.8 for most genes, while only a small proportion of genes showed extremely high tissue-specific (>0.8) or broad (<0.2) expression (supplementary fig. S5, Supplementary Material online). Kruskal–Wallis test was used to assess if Tau distribution differs across modules and we found that Tau values in different co-expression modules varied markedly (Kruskal–Wallis test: *P* < 0.001). A Wilcoxon rank-sum test with FDR corrections was used to compare the distribution of Tau in each module to the overall distribution. Generally, in somatic associated red and brown modules, genes showed relatively low Tau values, indicating low tissue specificity (supplementary fig. S6, Supplementary Material online). By contrast, we found relatively high and variable Tau values in most gonad-associated modules, except for the male gonad-associated green module and gonad-associated yellow module, which had relatively low Tau values, with median values at around 0.4 and 0.3, respectively (supplementary fig. S6, Supplementary Material online). Interestingly, we found that the yellow and green modules also showed relatively high intermodular connectivities, indicating that genes in these two modules showed also high connections with other tissues (fig. 2*D*).

**Fig. 4.**
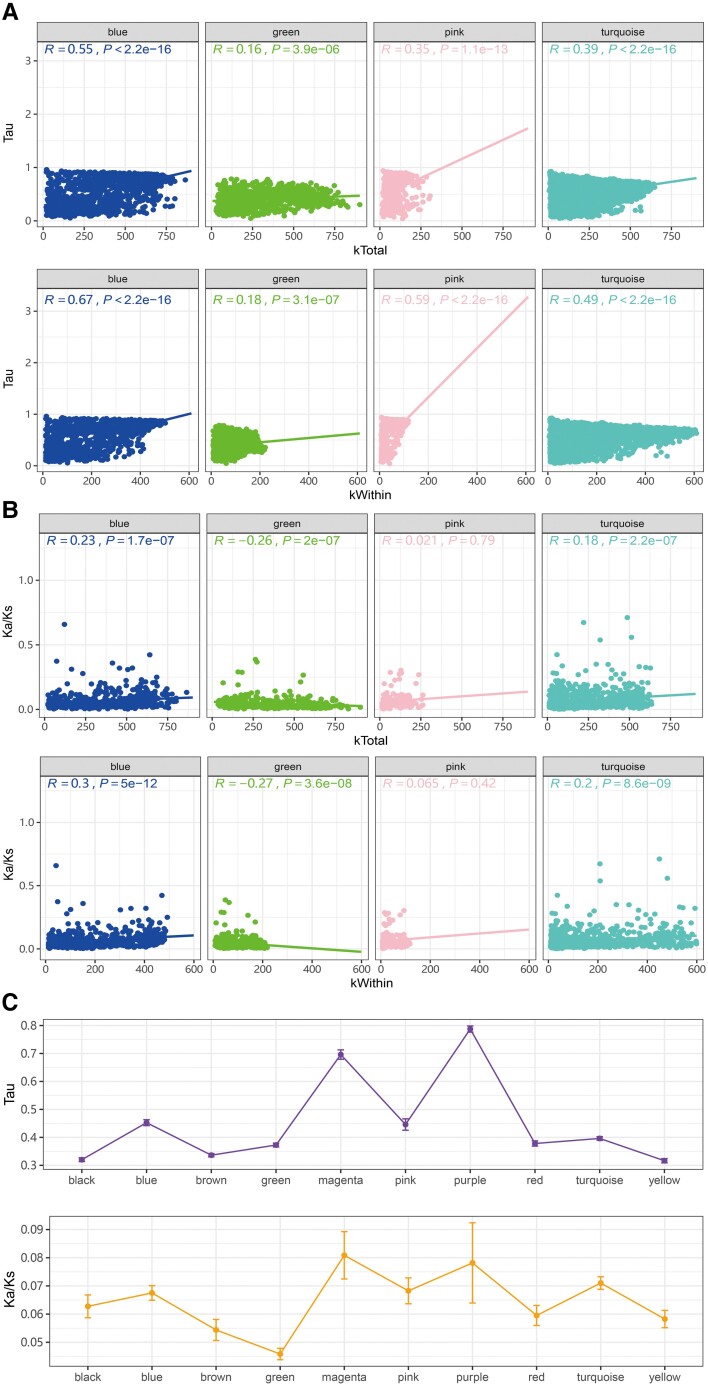
The relationship between network connectivity and tissue specificity, evolution rate. (*A*) The correlation between tissue specificity index (Tau) and total connectivity (kTotal), within module connectivity (kWithin) in four tissue-associated modules. The module name is shown on the top of each panel. (*B*) The correlation between evolutionary rate and total network connectivity (kTotal), within module connectivity (kWithin) for four tissue-associated modules. Spearman's correlation (*R*) and *P* values were shown on the top. (*C*) The trends of tissue specificity index (Tau) and evolutionary rate (Ka/Ks) in the co-expression modules. Average value (each dot) and standard error (error bar) was used for each module.

We further investigated the correlation between Tau and network connectivity using Spearman's rank sum test. We found positive correlation between whole network connectivity (kTotal) and tissue specificity (Tau) (Spearman's R = 0.24, *P* < 2.2E-16), and between intramodular connectivity (kWithin) and tissue specificity (Spearman's *R* = 0.34, *P* < 2.2E-16), but a weak correlation between intermodular connectivity (kOut) and tissue specificity (Spearman's *R* = −0.07, *P* = 6.433E-11). Moreover, we found significant positive correlation between tissue specificity Tau and kWithin, kTotal in most tissue-associated modules such as blue, pink, and turquoise modules, indicating that genes with high tissue specificity also presented high connection in the specific tissue type (fig. 4*A* and supplementary fig. S7A, Supplementary Material online). Additionally, the negative correlation between kOut and Tau was also observed in most modules except for blue, green, and yellow modules, where a positive correlation was observed (supplementary fig. S7*B*, Supplementary Material online).

We further investigated the tissue specificity for DEGs and DSGs in the co-expression network, mainly focusing on gonad-associated modules because of the low number of DEGs and DSGs in somatic tissues. Wilcoxon rank-sum test was used to assess differences in tissue specificity and network connectivity between DEGs and non-DEGs, and between DSGs and non-DSGs. In the male gonad-associated blue module, DSGs and DEGs presented significantly higher Tau values than non-DSGs and non-DEGs (fig. 3*C* and *D*). In the green module, DEGs also presented significantly higher Tau values than non-DEGs, whereas Tau values between DSGs and non-DSGs were not significantly different from each other (3*C* and *D*). However, in the pink module, Tau showed no significant difference between DEGs and non-DEGs, but DSGs presented slightly higher Tau values than non-DSGs (fig. 3*C* and *D*).

### Variation in the Rate of Sequence Evolution Across Co-Expression Modules

Kruskal–Wallis test, followed by Wilcoxon rank-sum test with FDR corrections, was used to test Ka/Ks differences across modules. Ka/Ks distribution also varied in different co-expression modules, with the male gonad-associated blue module and mantle-associated turquoise module presenting a significantly higher Ka/Ks than the overall values, and the green module presenting a significantly lower Ka/Ks than the overall values (supplementary fig. S8, Supplementary Material online). Spearman's rank sum test was used to measure the correlation between network connectivity and Ka/Ks, and between Tau and Ka/Ks. We found no significant correlation between general network connectivity (kTotal) and evolutionary rate (kTotal: Spearman's *R* = −0.0044, *P* = 0.78). However, when we investigated this relationship in each module, we found that genes in male gonad-associated blue module and mantle-associated turquoise module showed significantly positive correlation between network connectivities (both kTotal and kWithin) and evolutionary rates, while genes in the other male gonad-associated module (green module) showed significantly negative correlation between connectivities and evolutionary rates (fig. 4*B* and supplementary fig. S9*A*, Supplementary Material online). Most modules presented no significant correlation between intermodular connectivity and evolutionary rate (supplementary fig. S9*B*, Supplementary Material online).

Tau was positively correlated with Ka/Ks (Spearman's *R* = 0.17, *P* < 2.2E-16) in some tissue-associated modules. Similar to the correlation between connectivity and Ka/Ks, significant positive correlation between Tau and Ka/Ks was detected in blue and turquoise modules (supplementary fig. S10, Supplementary Material online). In spite of the lack of correlation in most modules, the Tau and Ka/Ks values showed similar trends across different modules (fig. 4*C*). Combined with the tissue specificity analysis above, it appears that genes in the male gonad-associated blue module and mantle-associated turquoise module with high intramodular connectivity and tissue-specificities also presented high evolutionary rates, while genes in the green module with high connections to outside the modules had a lower evolutionary rate.

Wilcoxon rank-sum test was used to assess differences in Ka/Ks between DEGs and non-DEGs, and between DSGs and non-DSGs. We also observed significant differences in evolutionary rate between DEGs and non-DEGs, and between DSGs and non-DSGs in the female and male gonad-associated modules (fig. 3*E* and *F*). In all three gonad-associated modules, DEGs presented significantly higher Ka/Ks than non-DEGs. Likewise, we found that DSGs in blue and pink modules also showed significantly higher Ka/Ks than non-DSGs, but such result was not detected in the male gonad-associated green module.

### Contrasting SNPs

We first retrieved SNPs for each sample separately and found that polymorphism in different tissues of the same individual was extremely low (fig. 1*C*). Thus, to retrieve sex-specific SNPs, we divided all the samples into female and male groups but merging the three tissues of the same individual together. We detected 750,790 total variants between male and female groups, of which 676,009 were SNPs. Of these, 252,858 SNPs were present in at least 80% of individual samples with a minimum quality score of 20. Filtered SNPs from male and female groups were analyzed using BayPass for contrast based on genotype counts, yielding 614 SNPs significantly contrasting between the two sexes (*P* < 0.001). Annovar merged the selected SNPs with the genome assembly annotation to identify the locations of each marker, specifying that of the 614 significantly contrasting SNPs, 381 were in exonic regions (supplementary table S13, Supplementary Material online). Finally, exonic SNPs from male and female groups were searched against a set of SNPs from a DNA pooled sequencing experiment of Mediterranean and Atlantic *R. philippinarum* populations (Smits et al. 2020) revealing that the two datasets contained 260 exonic SNPs in common. Genes containing contrasting SNPs are listed in supplementary table S9, Supplementary Material online, and some of them were also identified in DEGs, DSGs, or tissue-associated modules such as *ankyrin repeat domain-containing protein 17* (*ankrd17*), *double-strand-break repair protein rad21-like protein* (*rad21*), *folliculin* (*flcn*), *transcriptional regulator ATRX* (*atrx*). Functional enrichment indicated that genes containing contrasting SNPs were also involved in processes such as “mitochondrial transmembrane transport”, “protein localization to organelle”, and “chromatin remodeling” (supplementary table S11, Supplementary Material online).

## Discussion

In the present work, we sequenced and assembled a new long-read-based draft genome of the Manila clam *R. philippinarum*. Notably, this represents the first effort to sequence and assemble a wild (i.e., not inbred) specimen genome relying both on short and long-read data, and the first long-read genome assembly for this species. This genome assembly provides novel resources for Altantic populations, which has been observed to be genetically divergent to the Asian population, but very similar to the European population (Cordero et al. 2017). Additionally, the genome assembly allowed us to investigate tissue-specific gene expression and splicing patterns in *R. philippinarum*. Despite the increasing resources in terms of DNA and RNA sequences, most of the molecular pathways involved in tissue characterization are unknown in bivalves. Therefore, we also constructed a tissue-specific co-expression network whose analysis has been useful to identify candidate genes involved in the same biological processes. Genes showing the highest connection within a co-expression module are likely to have a central role in the corresponding module and are defined as “hub genes”. The analysis of hub genes has recently led to the identification of regulatory elements and biomarker targets for therapies (Grimes et al. 2019). We used high tissue specificity and high intramodular connectivity as proxies to identify networks of genes with tissue-specific functions, whereas low tissue specificity and high intermodular connectivity as a proxy of pleiotropy. We finally investigated the rate of protein evolution of genes in different modules and highlighted the complexity of gene regulation and sequence evolution in gonads.

### Both Differential Expression and Differential Splicing Shape Tissue-Specific Transcriptional Profiles in Bivalves

Different expression patterns between females and males have been investigated by several studies in gonochoric and sequential hermaphroditic bivalves, but mainly focused on the reproductive tissue alone (gonads), or across developmental stages (Ghiselli et al. 2012, 2018; Capt et al. 2018, 2019; Yue et al. 2018; Broquard et al. 2021). When extending the analyses of differential expression to multiple tissues, and adding the investigation of DS, we found that in *R. philippinarum* both DE and DS separate samples according to tissues (fig. 1). This suggests that both alternative splicing and DE have a central role in shaping tissue-specific transcriptional profiles in this species, and possibly in all bivalves. Additionally, both DE and DS analyses reveal a sex-specific transcriptional regulation in gonads, which leads male and female gonads to cluster separately from each other, a pattern that was not observed in somatic tissues. In other organisms, sex-biased genes and alternative splicing are reported to be responsible for most of the phenotypic differences between sexes (Parsch and Ellegren, 2013; Harrison et al. 2015; Ingleby et al. 2015; Lipinska et al. 2015; Dean and Mank, 2016; Rogers et al. 2021). In these cases, the majority of such genes are involved in sexual dimorphism and mating behavior. Such traits are absent in most bivalves (including *R. philippinarum*), and genes with gonad-specific and sex-specific transcriptional profiles are likely to be involved in sex determination, gonad specification, and gametogenesis. A functional annotation analysis of DE and DS genes, comparing male and female gonads and comparing gonad and somatic tissues, shows an enrichment of terms involved in reproduction, cell project organization, chromatin remodeling and DNA replication. These genes can help elucidating the molecular mechanism of gonad specification and sex differentiation in bivalves (see “Contrasting SNPs and Hub Genes Potentially Involved in Sex Determination and Mitochondrial Functions”).

### Co-Expression Network Analysis Reveals High Complexity in Gonad Gene Regulation

Although somatic tissues are usually associated with one or two co-expression modules, gonads are characterized by multiple, sometimes sex-specific, modules with different co-expression patterns (fig. 2), revealing a more complex gene regulation. Generally, genes in gonad-associated modules are characterized by higher tissue specificity compared with somatic tissue-associated modules (with the exception of the male gonad-associated green module, see below). Additionally, sequence evolution in each module follows a similar trend to tissue specificity, and such a relationship is particularly significant in gonad-associated blue and yellow modules (fig. 3*C* and supplementary fig. S10, Supplementary Material online). This trend is expected, because tissue-specific genes are less constrained compared with the pleiotropic genes, and they are usually characterized by higher sequence evolution (Dean and Mank, 2016; Mank et al. 2008; Meisel 2011).

Besides the co-occurrence of multiple co-expression networks in gonads, an additional level of complexity specifically characterizes male gonads, where different networks showed opposite trends of tissue specificity and rate of protein evolution. In more detail, the blue module shows a particularly high tissue specificity and rate of protein evolution; this is a pattern in line with the higher evolutionary rates of male-biased genes observed in a wide range of animals (Grath and Parsch, 2012; Parsch and Ellegren, 2013; Harrison et al. 2015). By contrast, the green module significantly deviates from what is observed in other gonad-specific networks: genes in this green module are indeed pleiotropic and constrained by a lower rate of protein evolution. Additionally, in contrast to other gonad-associated modules, there is no significant difference between DSGs and non-DSGs for tissue specificity and evolutionary rate in the green module, indicating that splicing may be underrepresented in highly pleiotropic genes. Such results reveal that a combination of genes with different transcription patterns, tissue specificity, and rate of protein evolution is required for male gonad differentiation.

When we looked at the functional annotation of genes belonging to the blue module, we found an enrichment of GO terms involved in reproduction. The fact that genes from this module are characterized by a faster sequence evolution is consistent with what is found in a wide range of species (Ellegren and Parsch, 2007; Grath and Parsch, 2012; Parsch and Ellegren, 2013; Harrison et al. 2015), in which male-biased genes are characterized by faster evolution. Interestingly, more than 70% of hub genes from this fast-evolving, highly tissue-specific module could not be annotated. This reveals that genes with a putative central role in male reproduction of *R. philippinarum* are mostly uncharacterized; it would be interesting to understand whether such genes show a male-biased transcription also in other bivalve or mollusc species, and investigate their evolution and role in male functions. Interestingly, most of hub genes from the other male-gonad-specific module (green module), are included in the KEGG BRITE category “cilium and associated proteins”, and they include sperm flagellum proteins and motile cilium-associated proteins. This module seems therefore to be majorly involved in the “structural” component of spermatogenesis, and it is not surprising that these genes are characterized by a slower evolution, as an improper formation of spermatozoa would likely undermine reproduction. Among the hub genes in this module, it is worth mentioning the presence of three out of five *tektin* genes. The *tektin* domain is also significantly enriched in the green modules. Tektins are cytoskeletal proteins associated with microtubules, and deficiency in these proteins are known to influence sperm motility and cause male infertility (Yan, 2009).

### Contrasting SNPs, Hub Genes and Domains Potentially Involved in Sex Determination and Mitochondrial Functions

Heteromorphic sex chromosomes are absent in bivalves, and sex determination is thought to be polygenic with the additional influence of environmental factors as potential triggers of sex changes (Breton et al. 2018; Dalpé et al. 2022). Identification of sex-specific SNPs is crucial for accurate sex diagnosis, breeding, and understanding of sex-determination mechanisms. In this study, we revealed 614 high-confidence contrasting SNPs between males and females, which provide potential genetic markers for sex identification in bivalves. Interestingly, we found that genes containing contrasting SNPs were overrepresented in the processes of protein targeting and protein localization to the mitochondrion. These genes included coiled-coil-helix-coiled-coil-helix domain-containing 2 (*cdchd2*), mitochondrial carrier protein Rim2, mitochondrial import inner membrane translocase subunit Tim16, and mitochondrial import inner membrane translocase subunit Tom22 (supplementary table S9, Supplementary Material online), with the latter three genes being involved in translocation of nuclear-encoded proteins into mitochondria (Herrmann and Neupert, 2013). Cdchd2 was found to be involved in diverse functions in model animals, including mediating oxidative phosphorylation, responding to hypoxic stress, regulating cell migration, and mitochondrial apoptosis (Kee et al. 2021). It has been proposed that DUI bivalves might have an unconventional sex determination/differentiation system that involves mitochondrial genomes and/or their products (proteins and/or RNAs), and this system may require an appropriate recognition/discrimination process between mitochondrial and nuclear factors (Breton et al. 2011, 2018; Ghiselli et al. 2013; Milani et al. 2013; Zouros, 2020). Although finding sex-specific SNPs in genes with mitochondrial function does not serve as direct evidence of the role of mitochondria in sex determination/differentiation in bivalves, it provides interesting candidate genes for testing such hypothesis in future experiments.

We also identified candidate genes and domains potentially associated with sex determination/differentiation mechanism in bivalves that are known to have a role in such processes in model animals. Among these, SRY-box transcription factor 30 (sox30), a putative homolog to mammal sex-determining gene *sry*, is a hub gene of the male gonad-associated blue module (supplementary table S9, Supplementary Material online). Sox30 has been found to be differentially expressed between females and males in many bivalve species (Ghiselli et al. 2012; Zhang et al. 2014; Capt et al. 2019); our analysis confirms a possible central role in sex determination/differentiation in *R. philippinarum*. For genes in the female gonad-associated pink module, zona pellucida and homeobox domain were significantly enriched. One interesting candidate gene with the homeobox domain is PBX homeobox 4 (*pbx4*). In our analyses, *pbx4* is a hub gene of the female gonad-associated pink module, and it is also differentially spliced between females and males (supplementary table S9, Supplementary Material online). The same gene in mammals has been found to be associated with gametogenesis (Wagner et al. 2001; Svingen and Koopman, 2007; Kawai et al. 2018). Also, *pbx* genes, which are characterized as hox gene co-activators, have been found to be associated with oogenesis, embryonic development, and germ cell maturation (Svingen and Koopman, 2007). Considering the female-specific transcription of *pbx4* in *R. philippinarum*, further analyses will be required to understand the role of this gene in bivalves. Finally, we found that MYCBP-associated protein expressed in testis 1-like (*maats1*) is the hub gene in the male gonad-associated green module. This gene was previously shown to be differentially expressed during spermatogenesis (Yukitake et al. 2002) and suggested to be a candidate gene influencing the sex transformation process in the fish *Monopterus albus* (Chi et al. 2017). This indicates a possible role of *maats1* in sex determination/differentiation in bivalves. Other hub genes such as spermatogenesis associated 17, testis-specific serine kinase 4, kelch-like family member 10 in three gonad-associated modules can be additional candidates involved in spermatogenesis, and therefore important in bivalve sex determination/differentiation system.

## Conclusions

In this study, we present a long-read-based de novo genome assembly of a Manila clam from the North American Pacific Coast and an extensive RNA-Seq multi-tissue (gonad, mantle, and adductor) analysis of 15 females and 15 females, providing insights into the role of DE and splicing in bivalve tissue identity. Although DS was largely overlapping with differential gene expression, it was preferentially involved in gonad functions. Co-expression network revealed complex gene regulation in gonads. Moreover, our data showed heterogeneity in sequence evolution for male gonad-associated genes in *R. philippinarum*. Apart from a gene set that follows the common observation that male-biased genes present high sequence evolution and remain mostly uncharacterized, we detected one additional set of male gonad-associated genes showing an extremely low sequence evolution, but high pleiotropy, and with a putative central role in male reproduction in *R. philippinarum*. Together, these results increase our understanding of the role of DE, DS, and sequence evolution of sex-specific genes. We also provide resourceful genomic data for further studies regarding sex diagnosis and breeding.

## Materials and Methods

A detailed Materials and Methods section with all parameter sets can be found in Supplementary Materials, Methods and Results, Supplementary Material online. A brief overview is described below.

### Sample Collection and Sequencing

Genomic DNA was extracted from a single male individual from the Puget Sound region (Pacific Northwest, USA) using only mantle tissue with the E.Z.N.A. Mollusc DNA Kit (Omega Bio-tek, Inc.). The PacBio library was prepared using a SMRTbell template preparation kit, and a 10–50 Kb size selection was performed using a BluePippin System. Two types of Illumina libraries were prepared: a “small insert” library (insert size ∼500 bp), and a “long insert” library (insert size ∼1,500 bp). To avoid as much as possible biases in library construction, we prepared multiple replicates for each library: nine replicates for the small insert library, and ten replicates for the large insert library. Replicates were indexed and pooled, and each pool was sequenced in one separated lane of an Illumina HiSeq 2,500 with 2 × 250 bp reads at the USC Genome Core facility, University of Southern California. The long-read libraries were sequenced on a PacBio RSII using a P6-C4 chemistry at the Genomics High-Throughput Facility, University of California, Irvine.

*Ruditapes philippinarum* specimens used for RNA-Seq were collected from the Northern Adriatic Sea, in the river Po delta region (Sacca di Goro, approximate GPS coordinates: 44°50′06′′*N*, 12°17′55′′E) during the spawning season (end of July). In total, 90 samples were obtained from three different tissues (adductor muscle, mantle, and gonad) of 15 males and 15 females. Total RNA was extracted with TRIzol, poly-A transcripts were isolated with magnetic beads and used as template for cDNA synthesis following the protocol as in Mortazavi et al. (2008) with modifications as in Ghiselli et al. (2012). RNA-sequencing was performed on Illumina HiSeq 2,500 platform with insert size of approximately 500 bp to generate 150 bp paired-end reads.

### Genome Assembly

Quality assessment and adaptor trimming of Illumina libraries were performed with Trimmomatic (Bolger et al. 2014) and FastQC. Genome size, heterozygosity, and duplication level were estimated using K-Mer Counter (Kokot et al. 2017), Genomescope 2 (Vurture et al. 2017) and kmercountexact.sh from the BBMap package (Bushnell, 2014) with different k-mer size.

Contig-level genome assembly was performed using PacBio reads and wtdbg2 (Ruan and Li, 2020). Contig correction and assembly heterozygosity reduction were performed running Hypo (Kundu et al. 2019) and purge_dups (Guan et al. 2020), respectively, for three consecutive times. Quality of the final version of the assembly was assessed with BUSCO (Seppey et al. 2019), redundans (Pryszcz and Gabaldón, 2016), and KAT (Mapleson et al. 2016). Possible contaminations in the assembly were identified and removed with Blobtools (Laetsch and Blaxter, 2017).

### Manila Clam Genome Comparison

Our assembly was aligned to a previously published *R. philippinarum* genome assembly (short-reads only) by Yan et al. (2019; GCA_009026015.1), that we named CRph, using the mummer package (Marçais et al. 2018). The dnadiff function was used to identify and classify alignable regions between the two assemblies.

### Genome Annotation

Transposable elements were annotated with RepeatModeler (Flynn et al. 2020) and MITE Tracker (Crescente et al. 2018). After removal of genes, tandem repeats and low copy number repeats, annotation of repeats was achieved running RepeatMasker (Tarailo-Graovac and Chen, 2009). Gene annotation was carried out using Maker (Cantarel et al. 2008). Three previously assembled transcriptomes of *R. philippinarum*, the Swiss-Prot database, and proteomes from *Crassostrea gigas* (GCF_902806645.1), *C. virginica* (GCF_002022765.2), *Lottia gigantea* (GCF_000327385.1), and *Octopus bimaculoides* (GCF_001194135.1) were used as external evidence. On these we trained SNAP (https://github.com/KorfLab/SNAP), Augustus (Stanke et al. 2008), genemark (Brůna et al. 2020), and Evidence Modeler (Haas et al. 2008). Predicted transcripts were annotated via Blastx (Altschul et al. 1990) against the full Swiss-Prot database, Pfam database and via InterProScan (Jones et al. 2014) with default options.

### Gene Expression and Co-Expression Analysis

The PE reads were processed with Trimmomatic (Bolger et al. 2014) to remove adaptors and low quality reads. Then, clean reads were mapped to the genome assembly using STAR (Dobin and Gingeras, 2015) in multiple 2-pass modes. FeatureCounts (Liao et al. 2014) was used to count the number of reads in the genomic features. Samples with a low number of reads and genes with a low expression level were filtered out using NOISeq (Tarazona et al. 2015). DE analysis was performed based on the filtered data in DESeq2 using both Wald test and Likelihood ratio test (Love et al. 2014). Genes with adjusted *P* values <0.05 and |log2(FoldChange)| > 1 were considered as DEGs. Tissue specificity for each gene based on Tau method was calculated using tspex (Camargo et al. 2020). Tissue specificity was estimated by Tau, an index for determining how specific or broad is gene expression. Tau ranges from 0 to 1, where 0 indicates broad expression across tissues and 1 indicates tissue-specific expression (Yanai et al. 2005). The co-expression network was constructed with Weighted Gene Co-expression Network Analysis (WGCNA) (Langfelder and Horvath, 2008). The network connectivity was retrieved from the co-expression network using the function *intramodularConnectivity* implemented in the WGCNA package. More in detail, for genes in the co-expression network, we measured the connectivity with genes in the same module (intramodular connectivity: kWithin), the connectivity with genes from different modules (intermodular connectivity: kOut) and its global connectivity (kTotal = kWithin + kOut). Therefore, kTotal, kWithin, and kOut in this tissue-specific co-expression network describe different properties: kTotal represents the total network connectivity and is the sum of kWithin and kOut; kWithin represents within module connectivity specific to one or multiple associated tissue types (specific connectivity); kOut represents the connection of one gene to the genes outside the module in the other tissue types (broad connectivity). Moreover, genes ranking in the top 5% of kWithin, representing high connection with the other genes in the module, were defined as the “hub” genes. The detailed parameters used in each program can be found in Supplementary Materials and Methods, Supplementary Material online.

### Differential Splicing Analysis

To understand the general pattern of splicing across tissues, intron excision ratio was calculated using Leafcutter (Li et al. 2018). A PCA plot based on the intron excision ratio was produced to visualize the general splicing patterns across tissues. For the pairwise DS analysis between sexes, and between pairwise tissues, we used exon-based limma package v3.42 (Ritchie et al. 2015), which presented good performances in DS analyses with large sample sizes (Mehmood et al. 2020; Merino et al. 2019). Genes with adjusted *P*-value <0.05 were considered differentially spliced (DS). The bam files generated from STAR were used for genome-guided transcriptome assembly in Stringtie (Pertea et al. 2016). SUPPA (Trincado et al. 2018) was used to measure seven alternative splicing events: skipping exon (SE), alternative 5′ splicing (A5), alternative 3′ splicing (A3), retained intron (RI), alternative first exon (AF), and alternative last exon (AL).

### Estimation of the Rate of Sequence Evolution

The protein coding sequences from the closely related species *Cyclina sinensis* (Family Veneridae) were retrieved from Wei et al (2020). Single-copy orthologs between *C. sinensis* and *R. philippinarum* were identified using OrthoFinder (Emms and Kelly, 2019). The orthologous protein sequences were aligned with Clustal Omega (Sievers and Higgins, 2018) and the nucleotide alignments were derived according to the protein alignments using PAL2NAL (Suyama et al. 2006). The protein evolutionary rate was estimated according to the ratio of non-synonymous to synonymous nucleotide changes (Ka/Ks), and it was calculated using KaKs_calculator2 (Wang et al. 2010).

### SNP Analysis

The quality of the reads from the male/female sequencing runs was assessed using the FastQC, before being mapped to the *R. philippinarum* genome assembly using Rsubread (Liao et al. 2019). The resulting BAM files were used for variant calling with Freebayes (Garrison and Marth, 2012) to retain only biallelic SNPs present in at least 80% of samples using Bcftools. Next, genotypes (in 0/1 format) were extracted from the two VCF files using the Genome Analysis ToolKit (GATK) (DePristo et al. 2011) and genotype counts by population were used as input for the BayPass (Gautier 2015). SNPs that were identified by BayPass as significantly contrasted between the male and female groups were then functionally annotated using Annovar (Wang et al. 2010). The effect of SNPs was predicted with SnpEff (Cingolani et al. 2012) and the PCA plot based on the SNPs across all samples was performed with SNPRelate (Zheng et al. 2012).

### Gene Set and Domain Enrichment

Gene Ontology (GO) analysis was performed for different sets of genes using topGO (Alexa 2021). The GO enrichment analysis was performed with Fisher's exact test, and REVIGO (Supek et al. 2011) was used to reduce redundancy in the enriched GO terms. Domain enrichment analysis was performed with Fisher's exact test in R using *fisher.test* function. The KEGG brite hierarchies for hub genes were performed in KAAS website (Moriya et al. 2007).

### Statistical Analysis

Kruskal–Wallis test followed by Dunn test with FDR correction were used to assess the pairwise difference in kTOtal, kWithin, kOut, Tau, and Ka/Ks. Wilcoxon rank-sum test was used to assess if there was difference for kWithin between DEG and no-DEGs, and between DSGs and no-DSGs. Wilcoxon rank-sum test with Holm–Bonferroni correction was used to compare module-specific kTotal, kWithin, kOut, Tau, and Ka/Ks to the overall values across all the modules. The correlation between pairwise two indexes was performed with Spearman's rank-sum test. All the tests and data visualization described above were performed in the Rstudio.

## Supplementary Material

evac171_Supplementary_Data

## Data Availability

PacBio and Illumina sequencing data as well as the genome assembly are deposited in the National Center for Biotechnology Information (NCBI: PRJNA807867). Genome annotation is available on https://doi.org/10.6084/m9.figshare.21069946.v5.
