# Supplementary material for: Multi-tissue RNA-Seq Analysis and Long-read-based Genome Assembly Reveal Complex Sex-specific Gene Regulation and Molecular Evolution in the Manila Clam
Source: Genome Biol Evol. 2022 Dec 12;14(12):evac171. doi: 10.1093/gbe/evac171 (PMC9803972; doi:10.1093/gbe/evac171)
Supplement: evac171_Supplementary_Data [file evac171_supplementary_data.zip › Revised_sup_materials&results.docx]

**Long-read-based genome assembly and multi-tissue RNA-Seq analysis reveal complex gene regulation and molecular evolution in the Manila clam**

Ran Xu^1,*,#^, Jacopo Martelossi^1,#^, Morgan Smits^2,#^, Mariangela Iannello^1,#^, Luca Peruzza^2^, Massimiliano Babbucci^2^, Massimo Milan^2^, Joseph P. Dunham^3,4^, Sophie Breton^5^, Liliana Milani^1^, Sergey V. Nuzhdin^3^, Luca Bargelloni^2^, Marco Passamonti^1,§^, Fabrizio Ghiselli^1,*,§^

**Supplementary Materials and Methods**

**Sample Collection and Library Preparation**

**Genome**

Genomic DNA (gDNA) was extracted from a single male individual from the Puget Sound region (Pacific Northwest, USA) using only mantle tissue with the E.Z.N.A. Mollusc DNA Kit (Omega Bio-tek, Inc.). The individual was opened, sexed, frozen in liquid nitrogen, and stored at -80ºC. Multiple DNA extractions were performed to obtain the amount of material required for both PacBio and Illumina libraries. The genomic DNA was quantified and its quality assessed using agarose gel electrophoresis, Nanodrop, and Bioanalyzer; before proceeding, the DNA had to meet the stringent PacBio requirements (i.e.: gDNA size >45 Kb, dsDNA, OD260/OD280 ratio of 1.8 to 2.0, OD260/OD230 ratio of ~2.0, does not contain insoluble material, does not contain RNA contamination, does not contain carryover contamination from the original organism/tissue such as polyphenols and polysaccharides). The PacBio library was prepared using a SMRTbell template preparation kit, and a 10-50Kb size selection was performed using a BluePippin System.

Two types of Illumina libraries were prepared: a “small insert” library (insert size ~500 bp), and a “long insert” library (insert size ~1,500 bp). To minimize batch effects and library preparation biases, we prepared multiple replicates for each library: 9 replicates for the small insert library, and 10 replicates for the large insert library. Replicates were indexed and pooled, and each pool was sequenced in one separated lane.

**Transcriptome**

*R. philippinarum* specimens used for RNA-Seq were collected from the Northern Adriatic Sea, in the river Po delta region (Sacca di Goro, approximate GPS coordinates: 44º50′06′′N, 12º17′55′′E) during the spawning season (end of July). The collected individuals were kept in the lab for 48 hours in aerated beakers containing artificial seawater—filtered reverse osmosis water with Red Sea Coral Pro aquariology sea salt (Red Sea Europe, Verneuilsur-Avre, France)—that was changed every 12 hours. Then, clams were opened, sexed by microscope inspection of gonadal tissue, flash-frozen in liquid nitrogen, and stored at −80°C until RNA extraction. Total RNA was extracted with TRIzol, poly-A transcripts were isolated with magnetic beads and used as template for cDNA synthesis following the protocol as in Mortazavi et al. (2008) with modifications as in Ghiselli et al. (2012). The selected insert size was approximately 500 bp. In total, after quality check, 90 samples were obtained from three different tissues (adductor muscle, mantle, and gonad) of 15 males and 15 females.

**Genome Sequencing and Assembly**

The short read libraries were sequenced on an Illumina HiSeq 2500 platform with 2x250 bp reads at the USC Genome Core facility, University of Southern California. The long read libraries were sequenced on a PacBio RSII using a P6-C4 chemistry at the Genomics High-Throughput Facility, University of California, Irvine.

**Short Read Trimming and Kmer-Based Genome Survey**

All Pair-end (PE) libraries were pre-processed with Trimmomatic v0.39 (Bolger et al., 2014) with parameters LEADING:3; TRAILING:3; SLINDINGWINDOW:4:15; MINLEN:36. Quality of filtered reads was checked with FastQC v0.11.8 and summarized with multiqc v1.9.

Genome size, heterozygosity and duplication level were estimated using all filtered short reads and K-Mer Counter v-3.1.1 (KMC; Kokot et al., 2017; parameters: kmer size=24-27-30, maximal value of a counter=5-E9). The resulting kmer histogram was uploaded to the online implementation of Genomescope 2 (Vurture et al., 2017). Additionally, kmercountexact.sh from the BBMap package (Bushnell, 2014) was used as a second tool for kmer-based genome size estimation (with a kmer size of 24, 27, and 30).

**Assembly Pipeline**

 To overcome high bivalve genome heterozygosity and produce an assembly as haploid as possible, we have based our assembly pipeline on up-to-date software which have proven to be cost-efficient and to perform well when dealing with highly heterozygous and non-model organisms (Guiglielmoni et al., 2021). First, a raw assembly was obtained using all PacBio reads with wtdbg2 (Ruan & Li, 2020) using the following parameters: -*p* 18 *-S* 2 *-g* 1.37 *-t* 0, as suggested by the developers in case of high error rate long sequences. Then, three consecutive rounds of haplotig removal and polishing were performed. In brief, first purge_dups (Guan et al., 2020) was used to identify duplicated contigs mapping back PacBio reads to the assembly and performing a whole genome self-alignment using Minimap2 (Li, 2018). Coverage cut-offs were automatically calculated, while the minimum fraction of haploid/bad/repetitive bases in a sequence (-f parameter) and the minimum alignment score (-a parameter) were decreased from respectively the default values of 0.8 to 0.7 and from 70 to 60 to increase its sensitivity. After that, both long reads and trimmed short reads were re-mapped against the reduced assembly and the resulting bam files used for polishing with Hypo (Kundu et al., 2019; genome size=1.37; approximate mean short reads coverage = 50). These steps were performed for three consecutive times, using BUSCO v.4 (Seppey et al., 2019) and the Metazoa odb10 core gene set as quality check. Finally, redundans (Pryszcz & Gabaldón, 2016) (default parameters), BUSCO, and KAT (Mapleson et al., 2016) were used to assess the quality of the final assembly.

**Contaminant Filtering**

To assess the presence of contaminants in the final assembly, we used Blobtools v. 2 (Laetsch & Blaxter, 2017). Each contig was blasted against the NCBI nt database with a stringent e-value of 1E-25 and annotated using the bestsum taxrule at the Phylum and species level. Only contigs annotated as Ascomycota, Bacteroidetes, Priapulida, and Zoopagomycota were systematically removed from the genome. Reads coverage and mapping statistics were calculated aligning both filtered short reads and long reads to the final version of the assembly with Minimap2 (Li, 2018). Mosdepth (Pedersen & Quinlan, 2018) was used to calculate per-base and median genome coverage excluding secondary alignments, optical duplicates, and low-quality reads (samtools flag -F 1796).

**Whole Genome Alignment and Structural Variant Detection**

Our assembly was aligned to the short-read-only, chromosome-level *R. philippinarum* genome assembly by Yan et al. (2019), called CRph genome, using the mummer V. 4 package (Marçais et al., 2018). We performed a first whole genome alignment (WGA) using the nucmer function with default parameters and summarizing results with the dnadiff function. A second WGA was performed with the aim to identify structural variations (SVs) between the two assemblies. For this analysis, we have adopted more stringent parameters to increase the specificity of the alignment (-l 100 -c 500) and allow the computation of all maximal matches regardless of their uniqueness (--maxmatch). The resulting delta file was then uploaded to Assemblytics (Nattestad & Schatz, 2016) for SVs calling with a required unique sequence length of 10,000 bp, a maximum variant size of 100,000 bp and a minimum variant size of 50 bp.

**Genome Annotation**

For Transposable element (TE) annotation we used a combination of *de novo* and homology-based approaches. In brief, RepeatModeler v2.0.1 (Flynn et al., 2020) with the LTR pipeline extension and MITE Tracker (Crescente et al., 2018) were used with default options for *de novo* mining of repeats. From resulting libraries, non-TE related genes were removed using blastx (E value 1E-10) against the predicted proteomes of *Crassostrea gigas* (GCF_902806645.1), *C. virginica* (GCF_002022765.2), *Lottia gigantea* (GCF_000327385.1), and *Octopus bimaculoides* (GCF_001194135.1) followed by ProtExcluder (Campbell et al., 2014). Tandem repeats were removed with the cleanup_tandem.pl script from the EDTA pipeline (Ou et al., 2019). Then, cleaned consensus libraries were merged with Mollusca repeats present in RepBase and redundancy removed using CD-HIT (Fu et al., 2012) following the 80-80 rule (80% similarity and coverage threshold). As a last step, the repeats library obtained was back-blasted against the assembly (Blastn, E value 1E-10; min query coverage 0.7; min identity 70%) and all repeats with less than 5 hits were removed to produce our final, clean set of TE consensus sequences. Genome annotation of repeats was achieved running RepeatMasker v4.1.0 (Tarailo‐Graovac & Chen, 2009) in sensitive mode (*-s* parameter).

Gene annotation was carried out using Maker/v3.01.03 (Cantarel et al., 2008) following a 3-step procedure: the first round was run by providing the repeat library, three previously assembled transcriptomes of *R. philippinarum*, the Swiss-Prot database, and the same molluscs proteomes used for TE annotation. In the second and third rounds of annotation, we used gene models produced from each previous Maker run to train gene predictors (i.e. SNAP/v2006-7-28, Augustus/v3.4.0 (Stanke et al., 2008), genemark/v4.58_lic (Brůna) and Evidence Modeler (Haas et al., 2008). Predicted proteins at the end of the third round were annotated via Blastx/v2.9.0+ (Altschul et al., 1990) against the full Swiss-Prot database, and Pfam database and via InterProScan/v 5.38-76.0 (Jones et al., 2014) with default options. Annotation was then included in the genome .gff file by using the “agat_sp_manage_functional_annotation.pl” tool (<https://github.com/NBISweden/AGAT>).

**RNA-Seq**

**Gene Expression and Co-Expression Analysis**

The PE reads were processed with Trimmomatic (Bolger et al., 2014) to remove adaptors and low quality reads with the following setting: LEADING:36 TRAILING:36 SLIDINGWINDOW:10:36 MINLEN:45. Then, clean reads were mapped to the genome assembly using STAR v2.7.7 (Dobin & Gingeras, 2015) in multiple 2-pass modes with the following settings: --outSAMattrIHstart 0 --outSAMstrandField intronMotif. FeatureCounts v2.0.2 (Liao et al., 2014) was used to count the number of reads in the genomic features. Samples with a low number of reads (<50,000) were removed and genes with a low expression level were filtered out using NOISeq v2.26.1 (Tarazona et al., 2015) with the following parameters: cpm = 1, cv.cutoff = 200. Differential expression analysis was performed based on the filtered data in DESeq2 v1.22.2 (Love et al., 2014). Wald test was used to retrieve DEGs in pairwise tissue comparisons in the same sex or pairwise sex comparison in the same tissue. Genes with adjusted p-values <0.05 and |log2(FoldChange)| > 1 were considered as differentially expressed genes (DEGs) in Wald test. Likelihood ratio test was used to investigate differentially expressed genes influenced by the interaction of sex and/or tissue, and genes with adjusted p-value < 0.05 were considered as DEGs. Tissue-specificity for each gene based on Tau method was calculated using tspex v0.6.1 (Camargo et al., 2020). Tissue-specificity was estimated by Tau, which is one of the most widely used methods for determining how specific or broad a gene is expressed and it ranges from 0 to 1, where 0 indicates broad expression across tissues and 1 indicates tissue-specific expression (Yanai et al., 2005). Therefore, Tau has been used as a proxy of pleiotropy by many studies (Dean & Mank, 2016; Mank et al., 2008; Rogers et al., 2021). The co-expression network was constructed with Weighted Gene Co-expression Network Analysis (WGCNA) v.1.66 (Langfelder & Horvath, 2008). To avoid the noise from lowly expressed genes in the co-expression network, a more stringent filtering was applied with cpm = 5 and cv.cutoff = 200 using NOISeq, and the vst transformed data from DESeq2 were used to build the co-expression network. The network connectivity was retrieved from the co-expression network using the function *intramodularConnectivity* implemented in the WGCNA package. More in detail, for genes in the co-expression network, we measured the connectivity with genes in the same module (intramodular connectivity: kWithin), the connectivity with genes from different modules (intermodular connectivity: kOut) and its global connectivity (kTotal=kWithin+kOut). Therefore, kTotal, kWithin, and kOut in this tissue-specific co-expression network describe different properties: kTotal represents the total network connectivity and is the sum of kWithin and kOut; kWithin represents within module connectivity specific to one or multiple associated tissue types (specific connectivity); kOut represents the connection of one gene to the genes outside the module in the other tissue types (broad connectivity). Moreover, genes ranking in the top 5% of kWithin, representing high connection with the other genes in the module, were defined as the “hub” genes.

**Differential Splicing Analysis**

To understand the general pattern of splicing across tissues, intron excision ratio was calculated using Leafcutter v0.2.9 (Li et al., 2018), an annotation-free tool for quantification of RNA splicing. A PCA plot based on the intron excision ratio was produced to visualize the general splicing patterns across tissues. For the pairwise differential splicing analysis between sexes, and between pairwise tissues, we used exon-based limma package v3.42 (Ritchie et al., 2015), which presented good performances in differential splicing analyses with large sample sizes (Mehmood et al., 2020; Merino et al., 2019). Genes with adjusted p-value <0.05 were considered differentially spliced (DS). The bam files generated from STAR were used for genome-guided transcriptome assembly in Stringtie v2.2.4 (Pertea et al., 2016), using the parameters: -F 2 -f 0.05. SUPPA v2.3 (Trincado et al., 2018) was used to measure seven alternative splicing events: skipping exon (SE), alternative 5’ splicing (A5), alternative 3’ splicing (A3), retained intron (RI), alternative first exon (AF) and alternative last exon (AL).

**Estimation of the rate of sequence evolution**

The protein coding sequences from the closely related species *Cyclina sinensis* (Family Veneridae) were retrieved from Wei et al. 2020,<https://doi.org/10.5061/dryad.44j0zpcb5>). Single-copy orthologs between *C. sinensis* and *R. philippinarum* were identified using OrthoFinder v2.5.1 with the default settings (Emms & Kelly, 2019). The orthologous protein sequences were aligned with Clustal Omega v 1.2.4 (Sievers et al., 2011) and the nucleotide alignments were derived according to the protein alignments using PAL2NAL (Suyama et al., 2006). The protein evolutionary rate was estimated according to the ratio of non-synonymous to synonymous nucleotide changes (Ka/Ks), and it was calculated using KaKs_calculator2 with the GMYN model (Wang et al., 2010).

**Gene Set and Domain Enrichment**

Gene Ontology (GO) analysis was performed for different sets of genes using topGO v2.44.0 (Alexa 2021). The GO enrichment analysis was performed with Fisher’s exact test based on the gene list with a minimum node size of 10 and a p-value cutoff of 0.01. REVIGO (Supek et al., 2011) was used to reduce redundancy in the enriched GO terms. Domain enrichment analysis was performed with Fisher’s exact test in R using *fisher.test* function, and a p-value of 0.01 was used for significance. Briefly, we counted the number of genes with Pfam domain annotations in the interested gene set and in the rest of gene set. Then fisher test was performed according to the contingency tables for each domain based on the number of genes containing such domain in the interested genes, the rest number of interested genes, the number of domains in the whole dataset, and the number of rest genes.

**SNP Analysis**

The quality of the reads from the male/female sequencing runs was assessed using the FastQC quality control tool v0.11.5, before being mapped to the *R. philippinarum* genome assembly using Rsubread v2.4.2 (Liao et al., 2019). Parameters for mapping male/female reads were: maxMismatches=10, nTrim5=5, nTrim3=6, unique=FALSE, nBestLocations=3.

  The resulting BAM files were used for variant calling with Freebayes v1.2.0 (Garrison & Marth, 2012), a tool for Bayesian haplotype-based genetic polymorphism discovery. Male/female population groups were analyzed with the following parameters: use-best-n-alleles=4, min-alternate-count=3, min-alternate-fraction=0.05, min-mapping-quality=20. The resulting VCF file was further filtered to retain only biallelic SNPs present in at least 80% of samples using Bcftools v1.11, according to the following criteria: min-alleles=2, max-alleles =2, type=snp, min-af=0.01, exclude-min-quality<20, exclude-max-missing>0.2.

Next, genotypes (in 0/1 format) were extracted from the two VCF files using the Genome Analysis ToolKit (GATK) v4.1.9.0 (DePristo et al., 2011) and genotype counts by population (n=2) were used as input for the BayPass package v2.2 (Gautier, 2015), a population genomics software primarily aimed at identifying genetic markers subjected to selection and/or associated to population-specific covariates. SNPs that were identified by BayPass as significantly contrasted (*P*< 0.001) between the male and female groups were then functionally annotated using Annovar (Wang et al., 2010). The effect of SNPs was predicted with SnpEff v4.3 (Cingolani et al., 2012) and the PCA plot based on the SNPs across all samples was performed with SNPRelate v1.26 (Zheng et al., 2012).

**Statistical Analysis**

Kruskal-Wallis test followed by Dunn test with FDR correction were used to assess the pairwise difference in kTOtal, kWithin, kOut, Tau, and Ka/Ks. Wilcoxon rank-sum test was used to assess if there was difference for kWithin between DEG and no-DEGs, and between DSGs and no-DSGs. Wilcoxon rank-sum test with Holm-Bonferroni correction was used to compare module-specific kTotal, kWithin, kOut, Tau, and Ka/Ks to the overall values across all the modules. The correlation between pairwise two indexes was performed with Spearman’s rank-sum test. All the tests and data visualization described above were performed in the R v4.1.4 ([https://www.R-project.org/](https://www.r-project.org/)).

**Supplementary Results and Discussion**

**Genome Sequencing, Assembly and Annotation**

In the present work we sequenced and assembled a new long-read-based draft genome of the Manila clam *Ruditapes philippinarum*. Notably, this represents the first effort to sequence and assemble a wild (i.e. not inbred) specimen relying both on short and long read data, and the first long read genome assembly for this species. In line with previous *R. philippinarum* sequencing projects (Mun et al., 2017; Yan et al., 2019), we estimated the genome size to range between 1.34 and 1.40 Gb with an extremely high level of heterozygosity depending on the kmer size and the utilized tool (3.7-4%) (Supplementary Table 3; Supplementary Figure 1). Interesting all kmer based estimation performed so far resulted much lower from what what was estimated by Feulgen method in the early century (1.97Gb; González-Tizón et al., 2000) Even if such features are common in molluscan genomes (Sun et al., 2021), to our knowledge our sample represents the most heterozygous mollusc genome sequenced so far. Such a high heterozygosity clearly represents one of the biggest obstacles in obtaining high quality assemblies (Sun et al., 2021), leading to high fragmentation or to strong collapsing of possible haplotigs. Our preliminary version of the assembly showed a greater than expected genome size of 1.61 Gb, a good contig N50 of 144 Kb, but a low level of BUSCO completeness (64.2%). These results are in line with recent published assembler benchmarks which show that wtdbg2, compared to other assemblers, has a good ability to collapse haplotigs but lower accuracy (Guiglielmoni et al., 2020). Our purging-polishing steps successfully increased all genome evaluation statistics without decreasing BUSCO completeness levels (Table 1). Overall BUSCO scores are in line with previously published bivalve genomes (95.3 % [Complete + Fragmented]) such as *Pinctada fucata* (Du et al., 2017) and *M. galloprovincialis* (Gerdol et al., 2020; Table 1). KAT analyses revealed a good collapsing of the assembly, with half of the total kmer present in the reads also present in the assembly, as expected by a heterozygous genome (Guiglielmoni et al., 2020; Table 1). Beside the 27 contigs annotated as clearly contaminants  because of a GC content and short read coverage that clearly deviates from the rest of the genome (Supplementary Figure 3), other 174 contigs were annotated as belonging to Chordata. However, they show the same GC content and coverage of Mollusca contigs (Supplementary Figure 4), at the species level eight of them were annotated as belonging to *R. philippinarum*, and 104 (60%) as *Pseudochaenichthys georgianus*, a fish with a distribution (Antarctic Peninsula and Scotia Sea) that does not overlap with our sampling area. For this reason they were not removed from the final version of the assembly. In summary, even if we cannot exclude the absolute absence of contamination in the final assembly, if present it should be in negligible amounts.

**Genome Comparison and Whole Genome Alignment**

We compared our assembly to the recently published *R. philippinarum* chromosome-level assembly by Yan et al (Yan et al., 2019), named CRph genome. Our assembly resulted in a significantly higher assembly size (1.41. Gb vs 1.12 Gb in Yan et al ^8^) and contig N50 (183 kb vs 28.1 kb in Yan et al., 2019), similar BUSCO completeness levels (92.7% in our assembly, 92.2% in Yan et al., 2019) and a higher level of duplications (8.6% in our assembly, only 1.9% in Yan et al., 2019) (Table 1). Out of the 15,908 contigs that composed our assembly, 15,781 (99.2%) had at least one alignment block to the CRph genome. In total, all alignment blocks represented 80% of our assembly (1,129,816,977 bp) (Supplementary Table 4). Of these, ~80% were aligned to assembled chromosomes of CRph genome, while the remaining 20% to unplaced or unlocalized scaffolds (*i.e* scaffolds that could not be placed in any chromosome). Interestingly, out of the 26,963 scaffolds that compose the CRph genome, of which 19 represent the assembled chromosomes, 10.4% (2,810) did not show any alignment block with our assembly (Supplementary Table 5), with all of them being classified as unlocalized scaffolds. Globally, 77.4% (868,865,079 bp) of the CRph genome got aligned, while one-to-one alignments cover 750,261,296 bp (Supplementary Table 4). Overall one-to-one aligned blocks had a mean nucleotide identity of 93% (Supplementary Table 4).

**References**

Mortazavi A, Williams BA, McCue K, Schaeffer L, Wold B. 2008. Mapping and quantifying mammalian transcriptomes by RNA-Seq. Nature Methods, 5: 621–628. DOI: 10.1038/nmeth.1226

Ghiselli F, Milani L, Chang PL, Hedgecock D, Davis JP, Nuzhdin S V., et al. 2012. De novo assembly of the Manila clam Ruditapes philippinarum transcriptome provides new insights into expression bias, mitochondrial doubly uniparental inheritance and sex determination. Molecular Biology and Evolution, 29: 771–786. DOI: 10.1093/molbev/msr248

Bolger AM, Lohse M, Usadel B. 2014. Genome analysis Trimmomatic: a flexible trimmer for Illumina sequence data. 30: 2114–2120. DOI: 10.1093/bioinformatics/btu170

Kokot M, Długosz M, Deorowicz S. 2017. KMC 3: counting and manipulating k-mer statistics. Bioinformatics, 33: 2759–2761. DOI: 10.1093/bioinformatics/btx304

Vurture GW, Sedlazeck FJ, Nattestad M, Underwood CJ, Fang H, Gurtowski J, et al. 2017. GenomeScope: fast reference-free genome profiling from short reads. Bioinformatics, 33: 2202–2204. DOI: 10.1093/bioinformatics/btx153

Bushnell B. 2014. BBMap: a fast, accurate, splice-aware aligner. Lawrence Berkeley National Lab.(LBNL), Berkeley, CA (United States).

Guiglielmoni N, Houtain A, Derzelle A, Van Doninck K, Flot J-F. 2021. Overcoming uncollapsed haplotypes in long-read assemblies of non-model organisms. BMC Bioinformatics, 22: 303. DOI: 10.1186/s12859-021-04118-3

Ruan J, Li H. 2020. Fast and accurate long-read assembly with wtdbg2. Nature Methods, 17: 155–158. DOI: 10.1038/s41592-019-0669-3

Guan D, McCarthy SA, Wood J, Howe K, Wang Y, Durbin R. 2020. Identifying and removing haplotypic duplication in primary genome assemblies. Bioinformatics, 36: 2896–2898. DOI: 10.1093/bioinformatics/btaa025

Li H. 2018. Minimap2: pairwise alignment for nucleotide sequences. Bioinformatics, 34: 3094–3100. DOI: 10.1093/bioinformatics/bty191

Kundu R, Joshua C, Sung W-K. 2019. HyPo: Super Fast & Accurate Polisher for Long Read Genome Assemblies. Biorxiv,

Seppey M, Manni M, Zdobnov EM. 2019. BUSCO: Assessing Genome Assembly and Annotation Completeness. In . 227–245. DOI: 10.1007/978-1-4939-9173-0_14

Pryszcz LP, Gabaldón T. 2016. Redundans: an assembly pipeline for highly heterozygous genomes. Nucleic Acids Research, 44: e113–e113. DOI: 10.1093/nar/gkw294

Laetsch DR, Blaxter ML. 2017. BlobTools: Interrogation of genome assemblies. F1000Research, 6: 1287. DOI: 10.12688/f1000research.12232.1

Pedersen BS, Quinlan AR. 2018. Mosdepth: quick coverage calculation for genomes and exomes. Bioinformatics, 34: 867–868. DOI: 10.1093/bioinformatics/btx699

Yan X, Nie H, Huo Z, Ding J, Li Z, Yan L, et al. 2019. Clam Genome Sequence Clarifies the Molecular Basis of Its Benthic Adaptation and Extraordinary Shell Color Diversity. iScience, 19: 1225–1237. DOI: 10.1016/j.isci.2019.08.049

Marçais G, Delcher AL, Phillippy AM, Coston R, Salzberg SL, Zimin A. 2018. MUMmer4: A fast and versatile genome alignment system. PLOS Computational Biology, 14: e1005944. DOI: 10.1371/journal.pcbi.1005944

Nattestad M, Schatz MC. 2016. Assemblytics: a web analytics tool for the detection of variants from an assembly. Bioinformatics, 32: 3021–3023. DOI: 10.1093/bioinformatics/btw369

Flynn JM, Hubley R, Goubert C, Rosen J, Clark AG, Feschotte C, et al. 2020. RepeatModeler2 for automated genomic discovery of transposable element families. Proceedings of the National Academy of Sciences, 117: 9451–9457. DOI: 10.1073/pnas.1921046117

Crescente JM, Zavallo D, Helguera M, Vanzetti LS. 2018. MITE Tracker: an accurate approach to identify miniature inverted-repeat transposable elements in large genomes. BMC Bioinformatics, 19: 348. DOI: 10.1186/s12859-018-2376-y

Campbell MS, Law M, Holt C, Stein JC, Moghe GD, Hufnagel DE, et al. 2014. MAKER-P: A Tool Kit for the Rapid Creation, Management, and Quality Control of Plant Genome Annotations. Plant Physiology, 164: 513–524. DOI: 10.1104/pp.113.230144

Ou S, Su W, Liao Y, Chougule K, Agda JRA, Hellinga AJ, et al. 2019. Benchmarking transposable element annotation methods for creation of a streamlined, comprehensive pipeline. Genome Biology, 20: 275. DOI: 10.1186/s13059-019-1905-y

Fu L, Niu B, Zhu Z, Wu S, Li W. 2012. Sequence analysis CD-HIT: accelerated for clustering the next-generation sequencing data. 28: 3150–3152. DOI: 10.1093/bioinformatics/bts565

Tarailo‐Graovac M, Chen N. 2009. Using RepeatMasker to Identify Repetitive Elements in Genomic Sequences. Current Protocols in Bioinformatics, 25 DOI: 10.1002/0471250953.bi0410s25

Cantarel BL, Korf I, Robb SMC, Parra G, Ross E, Moore B, et al. 2008. MAKER: An easy-to-use annotation pipeline designed for emerging model organism genomes. Genome Research, 18: 188–196. DOI: 10.1101/gr.6743907

Stanke M, Diekhans M, Baertsch R, Haussler D. 2008. Using native and syntenically mapped cDNA alignments to improve de novo gene finding. Bioinformatics, 24: 637–644. DOI: 10.1093/bioinformatics/btn013

Brůna T, Lomsadze A, Borodovsky M. 2020. GeneMark-EP+: eukaryotic gene prediction with self-training in the space of genes and proteins. NAR genomics and bioinformatics, 2(2): lqaa026.

Haas BJ, Salzberg SL, Zhu W, Pertea M, Allen JE, Orvis J, et al. 2008. Automated eukaryotic gene structure annotation using EVidenceModeler and the Program to Assemble Spliced Alignments. Genome Biology, 9: R7. DOI: 10.1186/gb-2008-9-1-r7

Altschul SF, Gish W, Miller W, Myers EW, Lipman DJ. 1990. Basic local alignment search tool. Journal of Molecular Biology, 215: 403–410. DOI: 10.1016/S0022-2836(05)80360-2

Jones P, Binns D, Chang H-Y, Fraser M, Li W, McAnulla C, et al. 2014. InterProScan 5: genome-scale protein function classification. Bioinformatics, 30: 1236–1240. DOI: 10.1093/bioinformatics/btu031

Dobin A, Gingeras TR. 2015. Mapping RNA‐seq Reads with STAR. Current Protocols in Bioinformatics, 51 DOI: 10.1002/0471250953.bi1114s51

Liao Y, Smyth GK, Shi W. 2014. featureCounts: an efficient general purpose program for assigning sequence reads to genomic features. Bioinformatics, 30: 923–930. DOI: 10.1093/bioinformatics/btt656

Tarazona S, Furí O-Tarí P, Turrà D, Pietro A Di, Jos´ MJ, Nueda J, et al. 2015. Data quality aware analysis of differential expression in RNA-seq with NOISeq R/Bioc package. Nucleic Acids Research, 43: 140. DOI: 10.1093/nar/gkv711

Love MI, Huber W, Anders S. 2014. Moderated estimation of fold change and dispersion for RNA-seq data with DESeq2. Genome Biology, 15: 550. DOI: 10.1186/s13059-014-0550-8

Camargo AP, Vasconcelos AA, Fiamenghi MB, Pereira GAG, Carazzolle MF. 2020. tspex: a tissue-specificity calculator for gene expression data. Preprint available at Research Square.

Yanai I, Benjamin H, Shmoish M, Chalifa-Caspi V, Shklar M, Ophir R, et al. 2005. Genome-wide midrange transcription profiles reveal expression level relationships in human tissue specification. Bioinformatics, 21: 650–659. DOI: 10.1093/bioinformatics/bti042

Dean R, Mank JE. 2016. Tissue specificity and sex-specific regulatory variation permit the evolution of sex-biased gene expression. American Naturalist, 188: E74–E84. DOI: 10.1086/687526

Mank JE, Hultin-Rosenberg L, Zwahlen M, Ellegren H. 2008. Pleiotropic constraint hampers the resolution of sexual antagonism in vertebrate gene expression. American Naturalist, 171: 35–43. DOI: 10.1086/523954

Rogers TF, Palmer DH, Wright AE. 2021. Sex-Specific Selection Drives the Evolution of Alternative Splicing in Birds. Molecular biology and evolution, 38: 519–530. DOI: 10.1093/molbev/msaa242

Langfelder P, Horvath S. 2008. WGCNA: An R package for weighted correlation network analysis. BMC Bioinformatics, 9 DOI: 10.1186/1471-2105-9-559

Li YI, Knowles DA, Humphrey J, Barbeira AN, Dickinson SP, Im HK, et al. 2018. Annotation-free quantification of RNA splicing using LeafCutter. Nature Genetics, 50: 151–158. DOI: 10.1038/s41588-017-0004-9

Ritchie ME, Phipson B, Wu D, Hu Y, Law CW, Shi W, et al. 2015. limma powers differential expression analyses for RNA-sequencing and microarray studies. Nucleic Acids Research, 43: e47–e47. DOI: 10.1093/nar/gkv007

Mehmood A, Laiho A, Venäläinen MS, Mcglinchey AJ, Wang N, Elo LL. 2020. Systematic evaluation of differential splicing tools for RNA-seq studies. Briefings in Bioinformatics, 21: 2052–2065. DOI: 10.1093/bib/bbz126

Merino GA, Conesa A, Ferná Ndez EA. 2019. A benchmarking of workflows for detecting differential splicing and differential expression at isoform level in human RNA-seq studies. Briefings in Bioinformatics, 20: 471–481. DOI: 10.1093/bib/bbx122

Pertea M, Kim D, Pertea GM, Leek JT, Salzberg SL. 2016. Transcript-level expression analysis of RNA-seq experiments with HISAT, StringTie and Ballgown. Nature Protocols, 11: 1650–1667. DOI: 10.1038/nprot.2016.095

Trincado JL, Entizne JC, Hysenaj G, Singh B, Skalic M, Elliott DJ, et al. 2018. SUPPA2: fast, accurate, and uncertainty-aware differential splicing analysis across multiple conditions. Genome Biology, 19: 40. DOI: 10.1186/s13059-018-1417-1

Wei M, Ge H, Shao C, Yan X, Nie H, Duan H, et al. 2020. Chromosome-Level Clam Genome Helps Elucidate the Molecular Basis of Adaptation to a Buried Lifestyle. iScience, 23: 101148. DOI: 10.1016/j.isci.2020.101148

Emms DM, Kelly S. 2019. OrthoFinder: Phylogenetic orthology inference for comparative genomics. Genome Biology, 20 DOI: 10.1186/s13059-019-1832-y

Sievers F, Wilm A, Dineen D, Gibson TJ, Karplus K, Li W, et al. 2011. Fast, scalable generation of high‐quality protein multiple sequence alignments using Clustal Omega. Molecular Systems Biology, 7: 539. DOI: 10.1038/msb.2011.75

Suyama M, Torrents D, Bork P. 2006. PAL2NAL: robust conversion of protein sequence alignments into the corresponding codon alignments. Nucleic Acids Research, 34: W609–W612. DOI: 10.1093/nar/gkl315

Wang K, Li M, Hakonarson H. 2010. ANNOVAR: functional annotation of genetic variants from high-throughput sequencing data. Nucleic Acids Research, 38: e164–e164. DOI: 10.1093/nar/gkq603

Alexa A, Rahnenfuhrer J. 2021. topGO: Enrichment Analysis for Gene Ontology. R package version 2.46.0

Supek F, Bošnjak M, Škunca N, Šmuc T. 2011. REVIGO Summarizes and Visualizes Long Lists of Gene Ontology Terms. PLoS ONE, 6: e21800. DOI: 10.1371/journal.pone.0021800

Liao Y, Smyth GK, Shi W. 2019. The R package Rsubread is easier, faster, cheaper and better for alignment and quantification of RNA sequencing reads. Nucleic Acids Research, 47: e47–e47. DOI: 10.1093/nar/gkz114

Garrison E, Marth G. 2012. Haplotype-based variant detection from short-read sequencing.arXiv preprint arXiv:1207.3907.

DePristo MA, Banks E, Poplin R, Garimella K V, Maguire JR, Hartl C, et al. 2011. A framework for variation discovery and genotyping using next-generation DNA sequencing data. Nature Genetics, 43: 491–498. DOI: 10.1038/ng.806

Gautier M. 2015. Genome-Wide Scan for Adaptive Divergence and Association with Population-Specific Covariates. Genetics, 201: 1555–1579. DOI: 10.1534/genetics.115.181453

Wang K, Li M, Hakonarson H. 2010. ANNOVAR: functional annotation of genetic variants from high-throughput sequencing data. Nucleic Acids Research, 38: e164–e164. DOI: 10.1093/nar/gkq603

Cingolani P, Platts A, Wang LL, Coon M, Nguyen T, Wang L, et al. 2012. A program for annotating and predicting the effects of single nucleotide polymorphisms, SnpEff SNPs in the genome of Drosophila melanogaster strain w 1118; iso-2; iso-3 DOI: 10.4161/fly.19695

Zheng X, Levine D, Shen J, Gogarten SM, Laurie C, Weir BS. 2012. A high-performance computing toolset for relatedness and principal component analysis of SNP data. Bioinformatics, 28: 3326–3328. DOI: 10.1093/bioinformatics/bts606.

Holt, C., 2011. MAKER2: An annotation pipeline and genome-database management tool for second-generation genome projects. BMC bioinformatics 12, 491. [doi.org/10.1186/1471-2105-12-491](https://doi.org/10.1186/1471-2105-12-491)
