# Supplementary material for: Multi-tissue RNA-Seq Analysis and Long-read-based Genome Assembly Reveal Complex Sex-specific Gene Regulation and Molecular Evolution in the Manila Clam
Source: Genome Biol Evol. 2022 Dec 12;14(12):evac171. doi: 10.1093/gbe/evac171 (PMC9803972; doi:10.1093/gbe/evac171)
Supplement: evac171_Supplementary_Data [file evac171_supplementary_data.zip › Sup_Figures_1-10.pdf]

Supplementary Figures

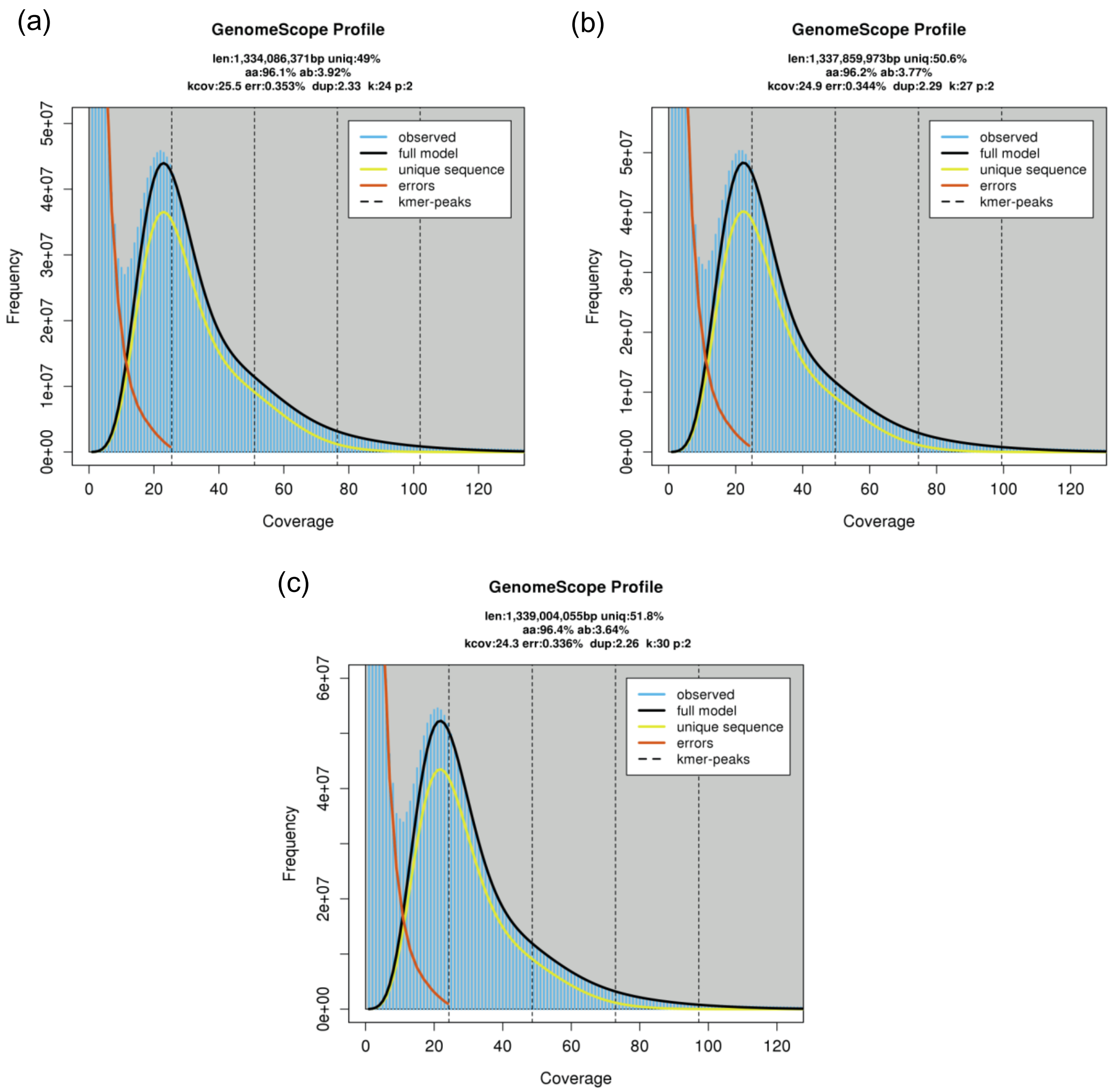

Supplementary Figure 1 Genome scope profiles with kmer size of 24 (**a**), 27 (**b**) and 30 (**c**).

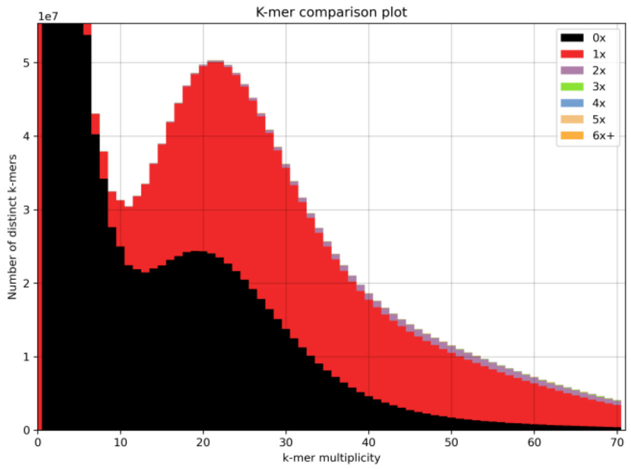

Supplementary Figure 2 KAT kmer spectra plot.

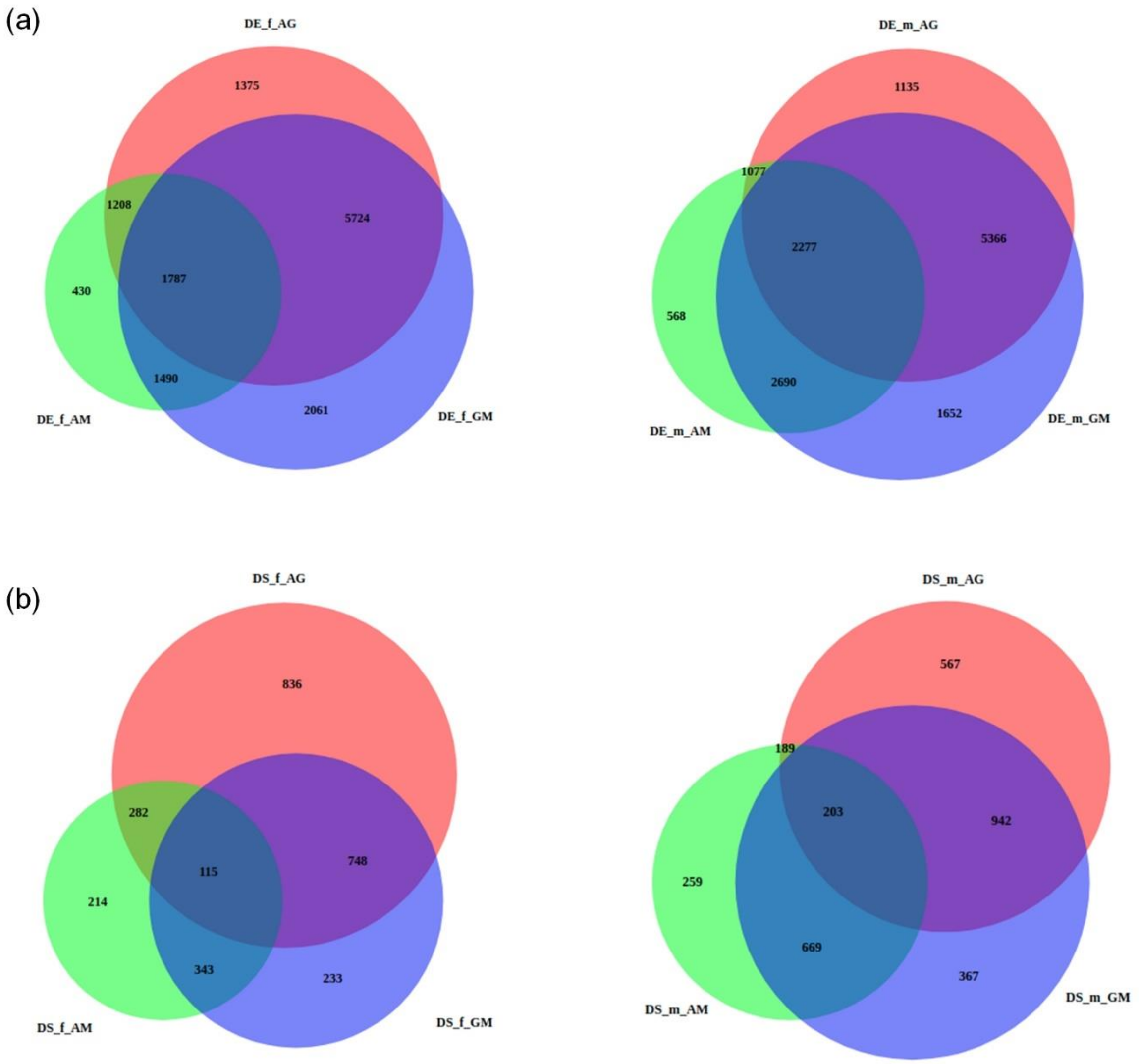

Supplementary Figure 3 Venn plot for differentially expressed (DE) genes and differentially spliced (DS) genes across tissues. Differentially expressed genes between pairwise genes in females (a) and males (b). Differentially spliced genes between pairwise genes in females (c) and males (d). f: female; m: male; A: adductor muscle; G: gonad; M: mantle.

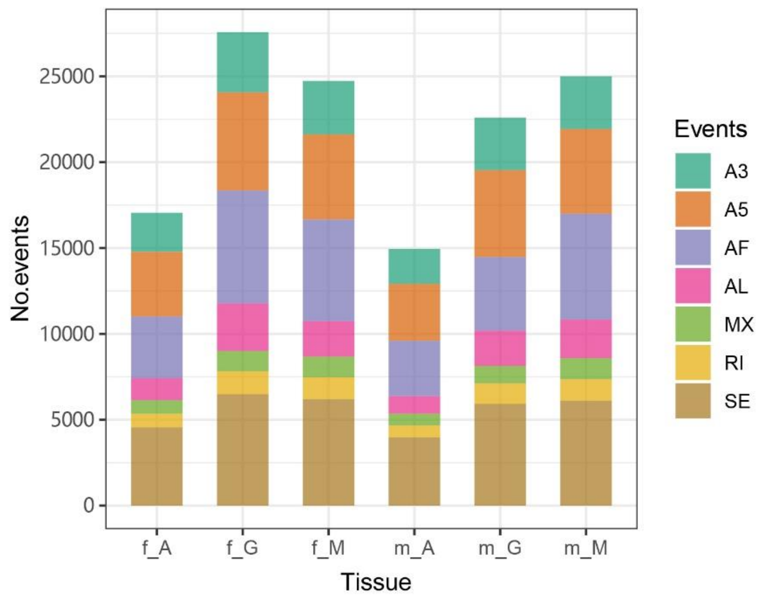

Supplementary Figure 4 The number of splice events in each tissue.

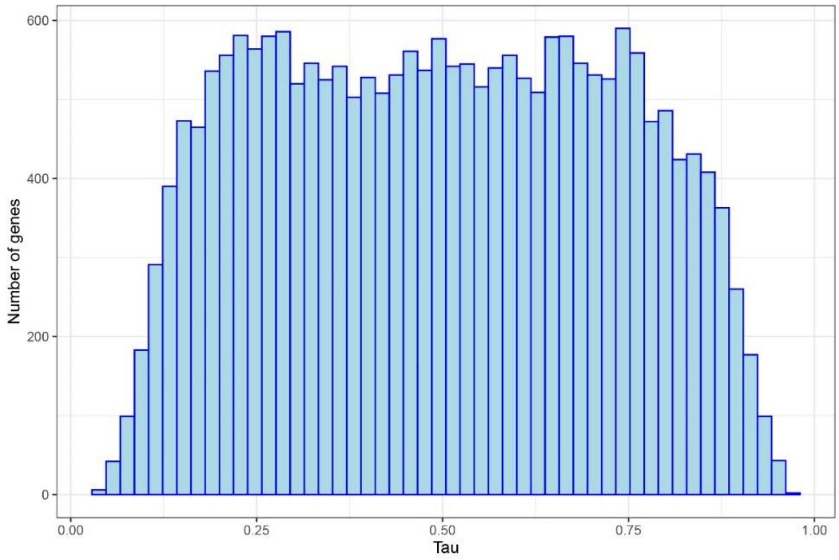

Supplementary Figure 5 Tissue specificity (Tau) index distribution.

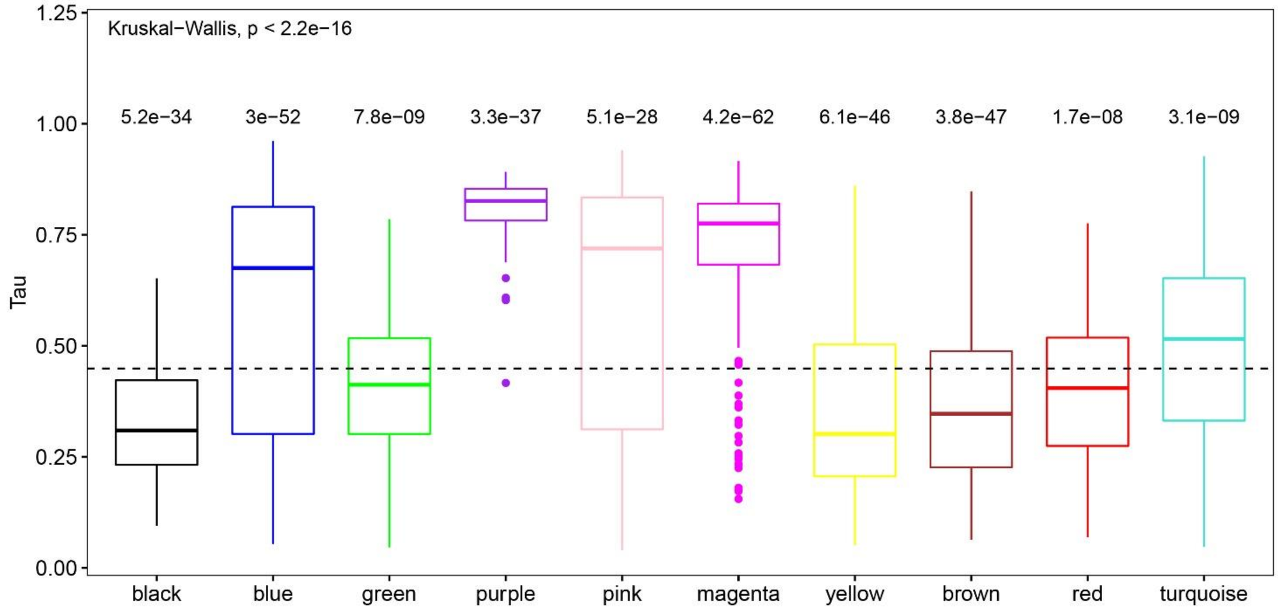

Supplementary Figure 6 The boxplot for tissue specificity in each module. Wilcoxon rank-sum test with FDR corrections was used to compare the distribution of Tau in each module to the overall distribution and the *P*-values were shown on the top of the boxplot. The dash line indicates the median of the overall distribution.

(a)

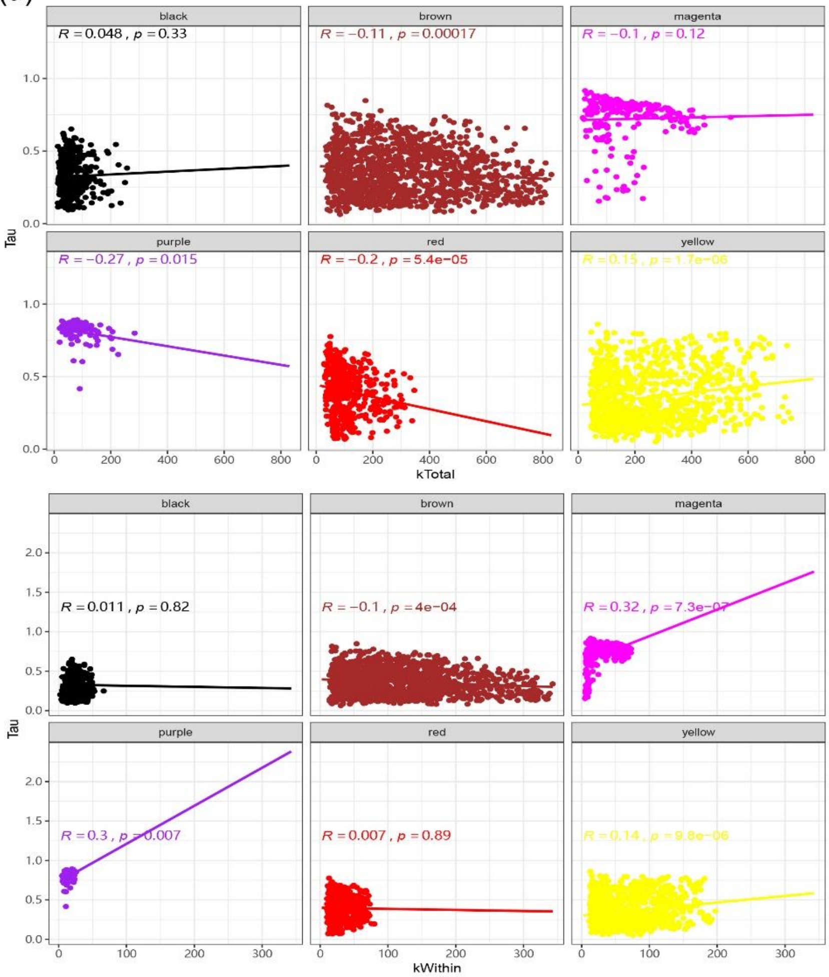
black
R = 0.048 , p = 0.33
brown
R = −0.11 , p = 0.00017
magenta
R = −0.1 , p = 0.12
purple
R = −0.27 , p = 0.015
red
R = −0.2 , p = 5.4e−05
yellow
R = 0.15 , p = 1.7e−06
Tau
kTotal
black
R = 0.011 , p = 0.82
brown
R = −0.1 , p = 4e−04
magenta
R = 0.32 , p = 7.3e−07
purple
R = 0.3 , p = 0.007
red
R = 0.007 , p = 0.89
yellow
R = 0.14 , p = 9.8e−06
Tau
kWithin

(b)

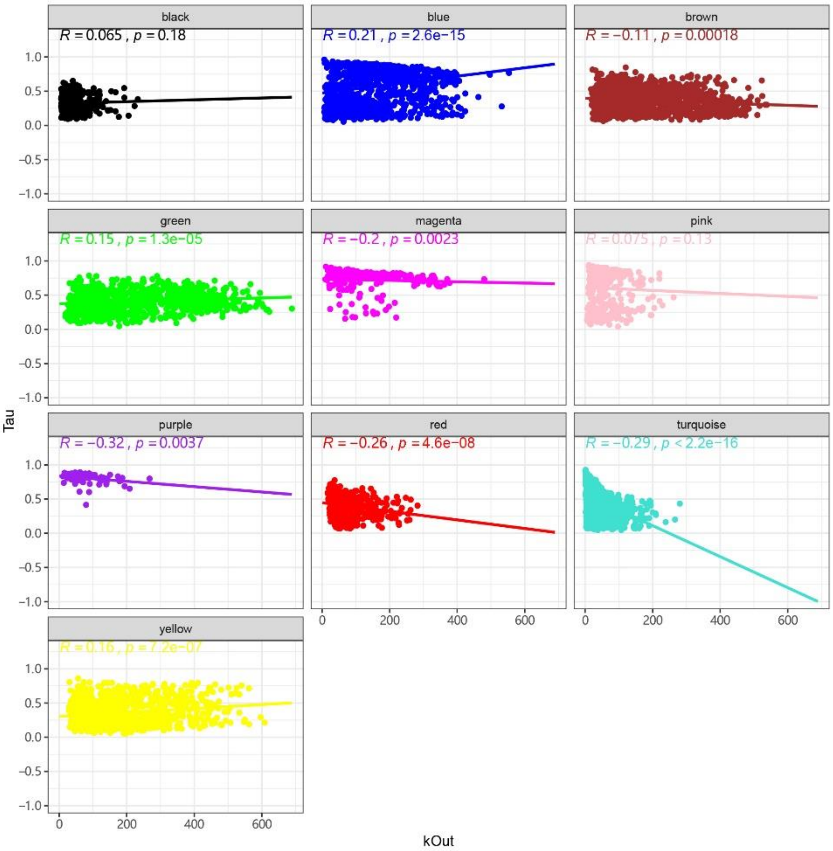
black
R = 0.065 , p = 0.18
blue
R = 0.21 , p = 2.6e−15
brown
R = −0.11 , p = 0.00018
green
R = 0.15 , p = 1.3e−05
magenta
R = −0.2 , p = 0.0023
pink
R = 0.075 , p = 0.13
purple
R = −0.32 , p = 0.0037
red
R = −0.26 , p = 4.6e−08
turquoise
R = −0.29 , p < 2.2e−16
yellow
R = 0.16 , p = 7.2e−07
Tau
kOut

Supplementary Figure 7 The correlation between Tau and connectivity. (a) The correlation between Tau and kTotal (top), and between Tau and kWithin (bottom). (b) The correlation between Tau and kOut.

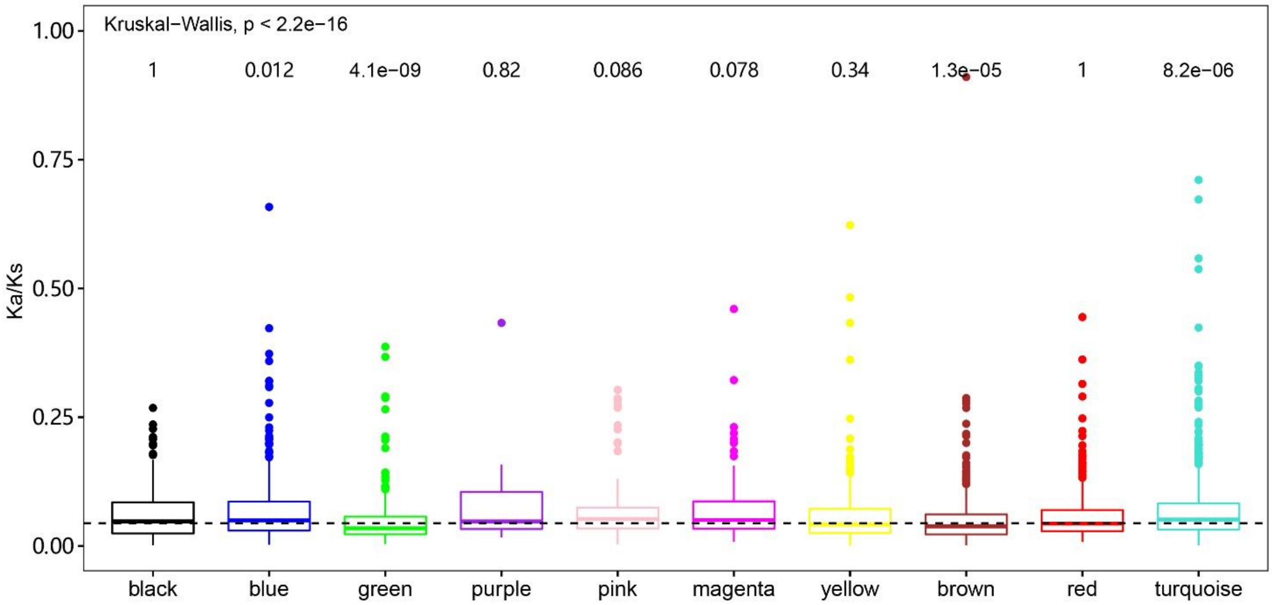

Supplementary Figure 8 The boxplot for Ka/Ks in each module. Wilcoxon rank-sum test with FDR corrections was used to compare the distribution of Tau in each module to the overall distribution and the *P*-values were shown on the top of the boxplot. The dash line indicates the median of the overall distribution.

(a)

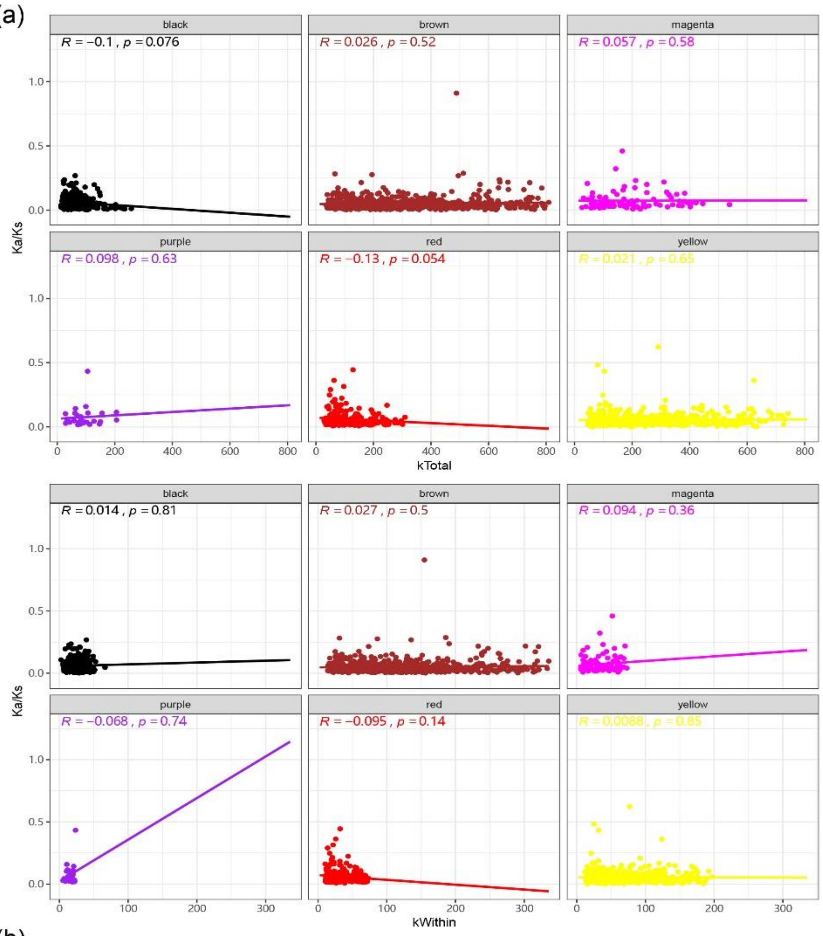
black
R = −0.1 , p = 0.076
brown
R = 0.026 , p = 0.52
magenta
R = 0.057 , p = 0.58
purple
R = 0.098 , p = 0.63
red
R = −0.13 , p = 0.054
yellow
R = 0.021 , p = 0.65
Ka/Ks
kTotal
black
R = 0.014 , p = 0.81
brown
R = 0.027 , p = 0.5
magenta
R = 0.094 , p = 0.36
purple
R = −0.068 , p = 0.74
red
R = −0.095 , p = 0.14
yellow
R = 0.0088 , p = 0.85
Ka/Ks
kWithin

(b)

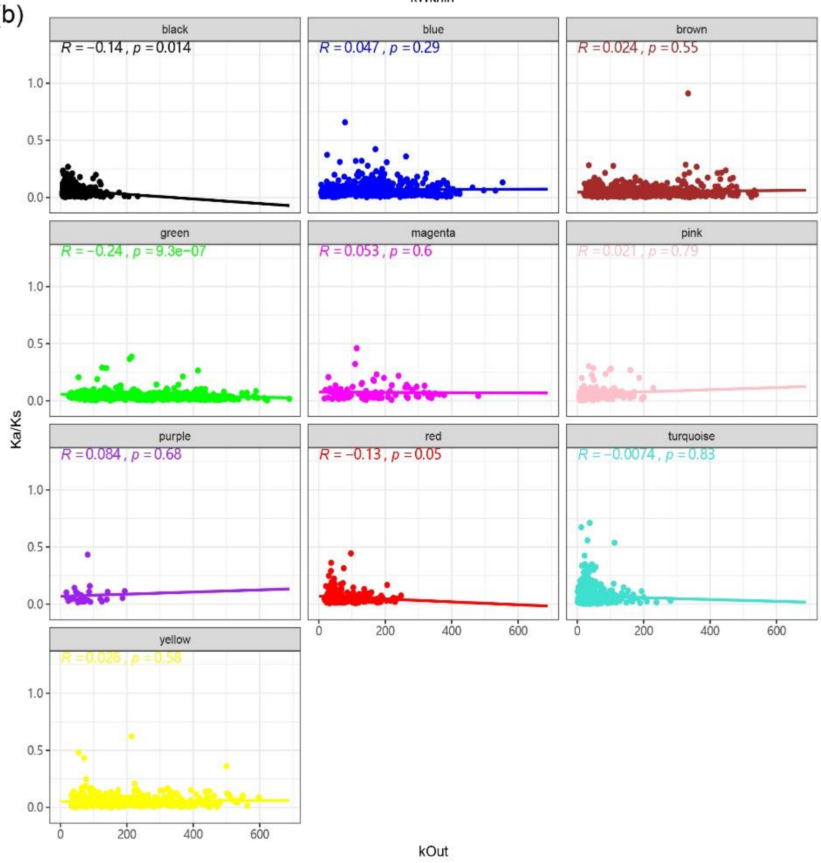
black
R = −0.14 , p = 0.014
blue
R = 0.047 , p = 0.29
brown
R = 0.024 , p = 0.55
green
R = −0.24 , p = 9.3e−07
magenta
R = 0.053 , p = 0.6
pink
R = 0.021 , p = 0.79
purple
R = 0.084 , p = 0.68
red
R = −0.13 , p = 0.05
turquoise
R = −0.0074 , p = 0.83
yellow
R = 0.026 , p = 0.58
Ka/Ks
kOut

Supplementary Figure 9 The correlation between Ka/Ks and connectivity. (a) The correlation between Ka/Ks and kTotal, and between Ka/Ks and kWithin. (b) The correlation between Ka/Ks and kOut.

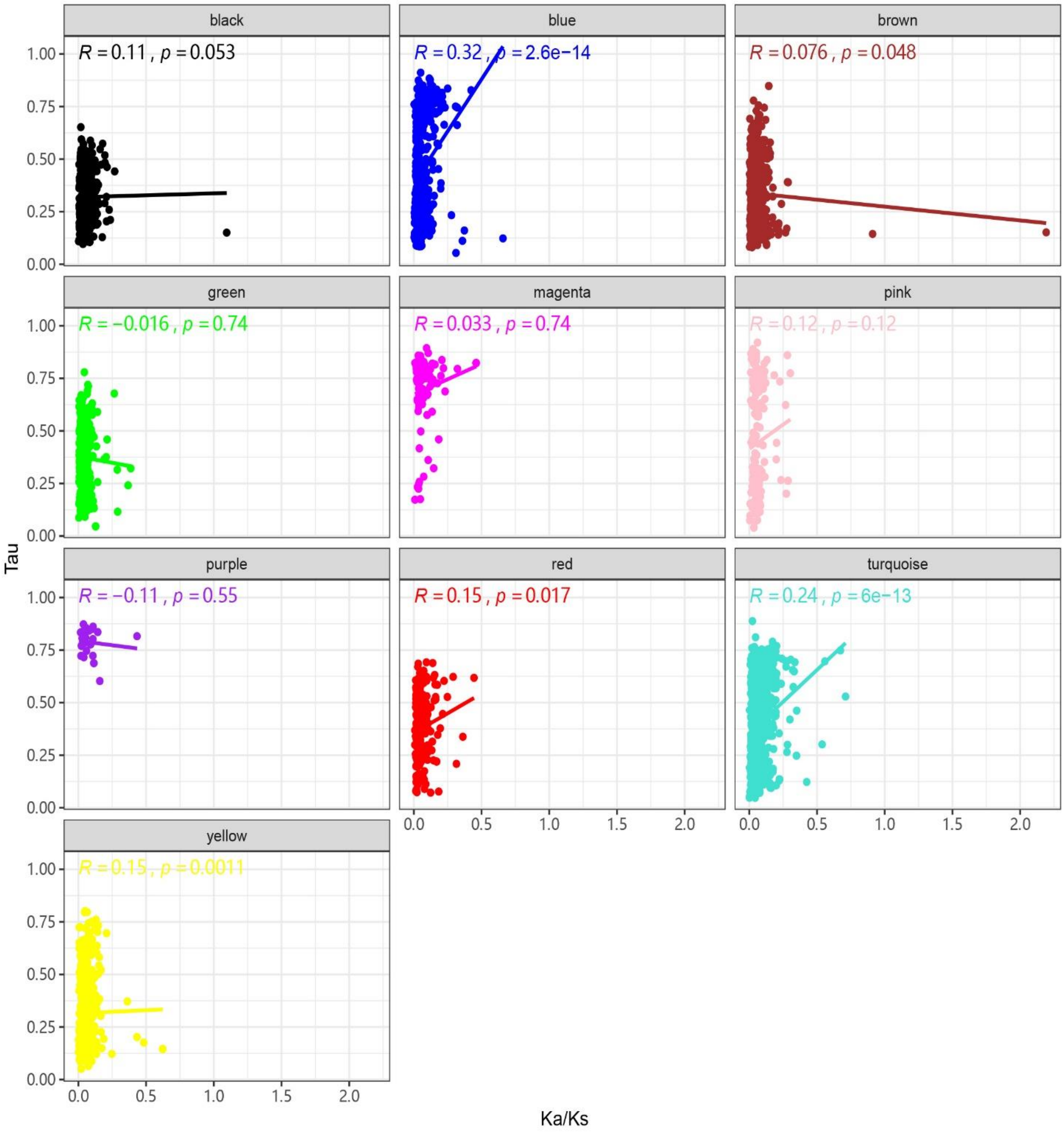

Supplementary Figure 10 The correlation between Tau and Ka/Ks in each module.
